# The Potential of Functional Near-Infrared Spectroscopy-Based Neurofeedback—A Systematic Review and Recommendations for Best Practice

**DOI:** 10.3389/fnins.2020.00594

**Published:** 2020-07-21

**Authors:** Simon H. Kohl, David M. A. Mehler, Michael Lührs, Robert T. Thibault, Kerstin Konrad, Bettina Sorger

**Affiliations:** ^1^JARA-Institute Molecular Neuroscience and Neuroimaging (INM-11), Jülich Research Centre, Jülich, Germany; ^2^Child Neuropsychology Section, Department of Child and Adolescent Psychiatry, Psychosomatics and Psychotherapy, Medical Faculty, RWTH Aachen University, Aachen, Germany; ^3^Department of Psychiatry, University of Münster, Münster, Germany; ^4^Brain Innovation B.V., Research Department, Maastricht, Netherlands; ^5^Faculty of Psychology and Neuroscience, Department of Cognitive Neuroscience, Maastricht University, Maastricht, Netherlands; ^6^School of Psychological Science, University of Bristol, Bristol, United Kingdom; ^7^MRC Integrative Epidemiology Unit, University of Bristol, Bristol, United Kingdom

**Keywords:** real-time data analysis, functional near-infrared spectroscopy, neurofeedback, systematic review, clinical translation, self-regulation, brain-computer interfacing

## Abstract

**Background:** The effects of electroencephalography (EEG) and functional magnetic resonance imaging (fMRI)-neurofeedback on brain activation and behaviors have been studied extensively in the past. More recently, researchers have begun to investigate the effects of functional near-infrared spectroscopy-based neurofeedback (fNIRS-neurofeedback). FNIRS is a functional neuroimaging technique based on brain hemodynamics, which is easy to use, portable, inexpensive, and has reduced sensitivity to movement artifacts.

**Method:** We provide the first systematic review and database of fNIRS-neurofeedback studies, synthesizing findings from 22 peer-reviewed studies (including a total of *N* = 441 participants; 337 healthy, 104 patients). We (1) give a comprehensive overview of how fNIRS-neurofeedback training protocols were implemented, (2) review the online signal-processing methods used, (3) evaluate the quality of studies using pre-set methodological and reporting quality criteria and also present statistical sensitivity/power analyses, (4) investigate the effectiveness of fNIRS-neurofeedback in modulating brain activation, and (5) review its effectiveness in changing behavior in healthy and pathological populations.

**Results and discussion:** (1–2) Published studies are heterogeneous (e.g., neurofeedback targets, investigated populations, applied training protocols, and methods). (3) Large randomized controlled trials are still lacking. In view of the novelty of the field, the quality of the published studies is moderate. We identified room for improvement in reporting important information and statistical power to detect realistic effects. (4) Several studies show that people can regulate hemodynamic signals from cortical brain regions with fNIRS-neurofeedback and (5) these studies indicate the feasibility of modulating motor control and prefrontal brain functioning in healthy participants and ameliorating symptoms in clinical populations (stroke, ADHD, autism, and social anxiety). However, valid conclusions about specificity or potential clinical utility are premature.

**Conclusion:** Due to the advantages of practicability and relatively low cost, fNIRS-neurofeedback might provide a suitable and powerful alternative to EEG and fMRI neurofeedback and has great potential for clinical translation of neurofeedback. Together with more rigorous research and reporting practices, further methodological improvements may lead to a more solid understanding of fNIRS-neurofeedback. Future research will benefit from exploiting the advantages of fNIRS, which offers unique opportunities for neurofeedback research.

## Introduction

Functional near-infrared spectroscopy (fNIRS) is a growing functional neuroimaging technique that exploits the principles of near-infrared (NIR) spectroscopy and brain hemodynamics. Human tissues, including brain tissue, are relatively transparent to light in the NIR range (650–1,000 nm). If NIR light is directed onto the surface of the head most of the light scatters within the underlying tissue, while some of the light is absorbed by pigmented compounds (chromophores). The main chromophore hemoglobin (red blood cells transporting oxygen) absorbs and attenuates the NIR light, and the absorption spectrum of hemoglobin is dependent on the oxygenation level, i.e., oxy-(HbO) > 800 nm and deoxyhemoglobin (HbR) <800 nm. This principle is utilized by fNIRS to detect relative changes of HbO and HbR levels and thereby indirectly estimating brain activation in the underlying brain tissue via optical sensors placed on the surface of the head (see Ferrari and Quaresima, 2012; Pinti et al., 2018b).

Compared to other neuroimaging modalities, such as functional magnetic resonance imaging (fMRI), electroencephalography (EEG), and magnetoencephalography (MEG), fNIRS shows both advantages and disadvantages. First, the spatial and temporal resolution of fNIRS lies between fMRI and EEG. It provides higher spatial resolution (between 2 and 3 cm) than EEG and potentially higher temporal resolution than fMRI, due to a higher sampling rate. The depth of fNIRS measurements is restricted to neocortical brain regions (Pinti et al., 2018b). FNIRS also has a lower spatial resolution and lower signal-to-noise ratio compared to fMRI (Cui et al., 2011). However, the practicability of fNIRS is a major advantage over fMRI: it is easier to use, portable, safe, nearly silent, inexpensive, and requires little setup time. Moreover, fNIRS measurements tolerate more head motion compared to EEG and fMRI measurements. This makes it possible to use fNIRS in more naturalistic environments/situations (e.g., allowing neural activity to be recorded during overt speech, movement, and direct interaction with another person). Furthermore, it permits populations to be investigated that are more likely to show head motion (e.g., neurological or psychiatric patients or infants) and situations that do not allow fMRI measurements (e.g., participants with ferromagnetic implants or claustrophobia). For recent reviews on the use of fNIRS in neuroscience see Pinti et al. (2018b) and Quaresima and Ferrari (2019).

Given the advantages of fNIRS over other neuroimaging modalities, this technique has been increasingly used as a tool for neurofeedback (Ehlis et al., 2018). During neurofeedback training, participants are trained to self-regulate their brain activity, generally with the ultimate goal of changing behavior or cognitive/emotional functions (for reviews see Thibault et al., 2016; Sitaram et al., 2017; Paret et al., 2019). **Figure 2** (upper part) shows a typical fNIRS-neurofeedback setup. Changes in HbO, HbR, or total hemoglobin (tHb) are assessed via optodes placed on the participants' heads covering a certain brain region of interest and are usually fed back to the subject in the form of visual representations. Individuals can then use this feedback information to learn successful self-regulation of brain activity and ideally transfer this skill to daily life. Successful neurofeedback training usually requires several neurofeedback sessions [1–5 sessions for fMRI-neurofeedback and up to 30 sessions for EEG-neurofeedback (see Thibault et al., 2016)], which is costly and difficult to perform with fMRI.

To date, there has been no comprehensive *systematic* review of fNIRS-neurofeedback studies. The available reviews are either not systematic or are *selective* (not covering all published fNIRS-neurofeedback work). Some reviews focus more on general aspects of fNIRS-based brain-computer interfacing (e.g., Naseer and Hong, 2015; Thibault et al., 2016; Ehlis et al., 2018). For example, a recent review by Ehlis et al. (2018) reviewed several of their own studies alongside a few other experiments. They concluded that fNIRS-neurofeedback training can enable participants to regulate their hemodynamic responses deliberately and that this training may induce changes in brain functions over time. Further, Ehlis et al. (2018) conclude that if future studies confirmed initial findings, fNIRS-neurofeedback may become a complementary or even alternative treatment option for neuropsychiatric disorders.

The present systematic review is divided into five stand-alone sections. We (1) synthesize information about training protocols; (2) provide an overview of the methods used for online signal-processing to calculate the feedback signal; (3) critically evaluate the quality of published studies including experimental designs, reporting (Tufanaru et al., 2017; Ros et al., 2020), and statistical power; (4) assess and discuss the effectiveness of fNIRS-neurofeedback to regulate and induce pre-post changes in brain activity; and (5) assess and discuss its effectiveness in inducing changes in behavioral/cognitive/emotional^1^ outcome measurements in healthy and pathological populations and also review the clinical potential of fNIRS-neurofeedback. We finish the review with a discussion arising from the findings of the five sections and also touch on the future of fNIRS-neurofeedback research.

## Methods

The study protocol for this systematic review was registered on *PROSPERO* and can be accessed at https://www.crd.york.ac.uk/PROSPERO/display_record.php?RecordID=141049. Data from this systematic review are available at the Open Science Framework (see https://osf.io/hnxfq/). We followed the Preferred Reporting Items for Systematic Reviews and Meta-Analyses (PRISMA) guidelines (Moher et al., 2009).

### Search Strategy

We searched the following electronic bibliographic databases for studies published up until 3 July 2019: PubMed/MEDLINE, Web of Knowledge/Web of Science, Scopus, and EMBASE. Additional searches were conducted using the Real-time Functional Imaging and Neurofeedback (rtFIN) database (rtfin.org), Cochrane Reviews library database (cochranelibrary.com), Clinicaltrials.gov, scholar.google.de, and preprint servers: biorxiv.org, arxiv.org, psyarxiv.com, medrxiv.org, and osf.io. The following search terms were used: (“functional near infrared spectroscopy” OR “fNIRS” OR “near infrared spectroscopy” OR “NIRS”) AND (“real-time” OR “real time” OR “neurofeedback” OR “biofeedback” OR “Brain-Computer Interface” OR “Brain computer interface” OR “BCI” OR “Brain-Machine Interface” OR “Brain Machine Interface” OR “BMI”). More details of the search strategy can be found in the protocol or via the following link: https://www.crd.york.ac.uk/PROSPEROFILES/141049_STRATEGY_20190703.pdf.

### Study Selection

**Figure 1:**
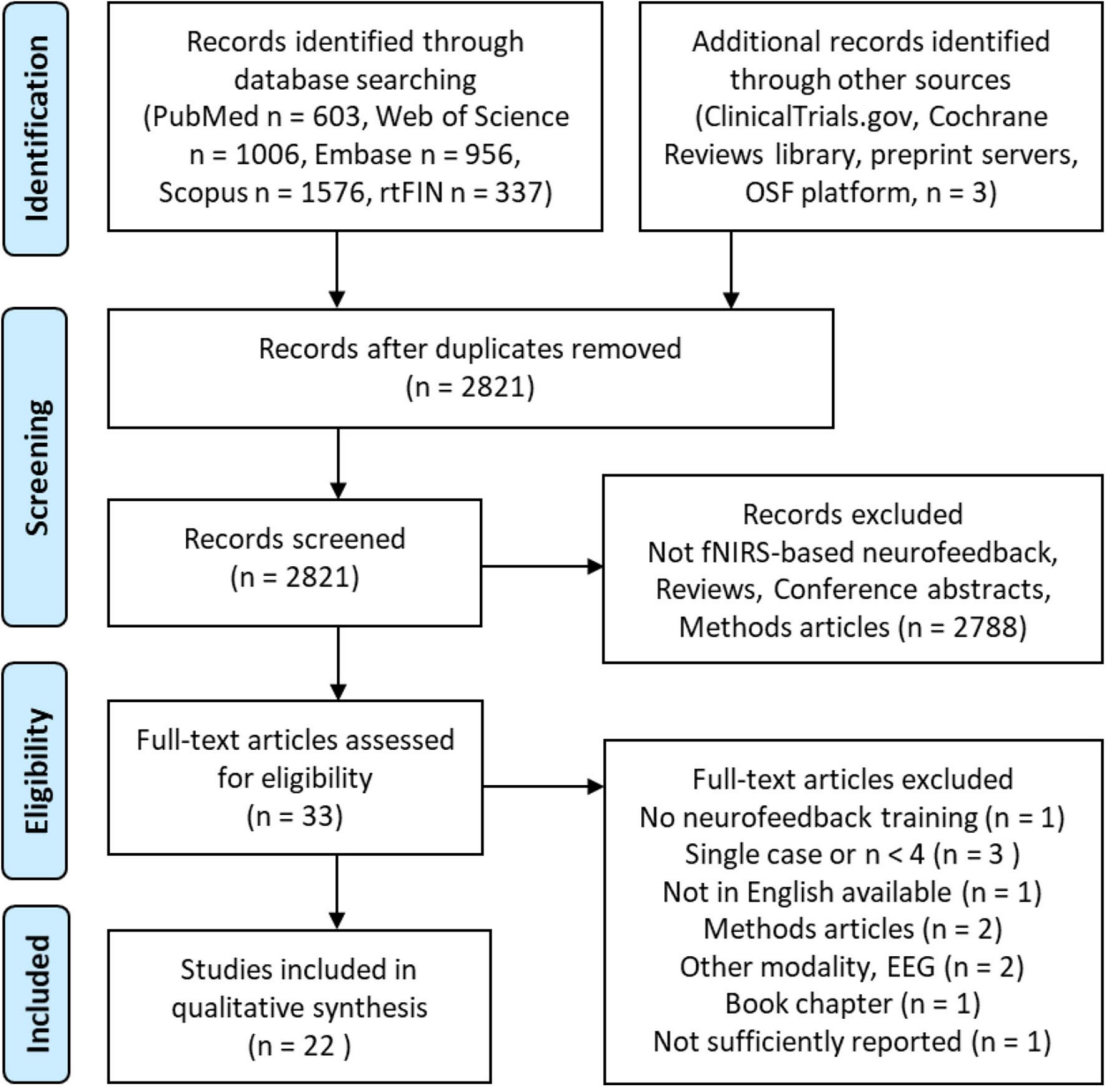
Search decision flow diagram according to preferred reporting items for systematic reviews and meta-analyses (PRISMA; Moher et al., 2009).

We included all published articles with study designs that applied fNIRS-neurofeedback training to regulate brain activity and/or *behavior* in healthy or patient populations. The field is very young and to date no randomized controlled trials have been published. Hence, we applied rather loose inclusion criteria, also including non-controlled, pilot, feasibility, and proof-of-concept studies involving at least four participants. Studies applying fNIRS only for the purpose of brain-based communication or control of devices were excluded. After removing duplicates, the titles and abstracts of 2,821 articles were screened and the full-text of the remaining 33 articles was retrieved and assessed for eligibility. Twenty-two studies met the inclusion criteria and were considered in the qualitative synthesis (see Figure 1), involving a total of 441 participants (337 healthy participants and 104 patients).

### Data Extraction and Analysis

A spreadsheet was used to document data extracted from the studies. Extracted data included: information about study population and study design, details of the neurofeedback protocol and control conditions; methods used for online signal-processing to calculate the feedback information, and outcomes of the neurofeedback training categorized into behavioral and neural effects within and independent of targeted brain regions (see Supplementary Material). After finishing the data extraction, the spreadsheet was sent to all corresponding authors of the included studies to ask for corrections. Fifteen of 22 authors replied and either approved the data extraction or sent minor corrections. We also gave authors the opportunity to comment on a preprint version of the manuscript, which was uploaded at the Open Science Framework (see https://osf.io/hnxfq/) before submission. Further methodological details will be provided in the respective sections.

## Results and Discussion

**Figure 2:**
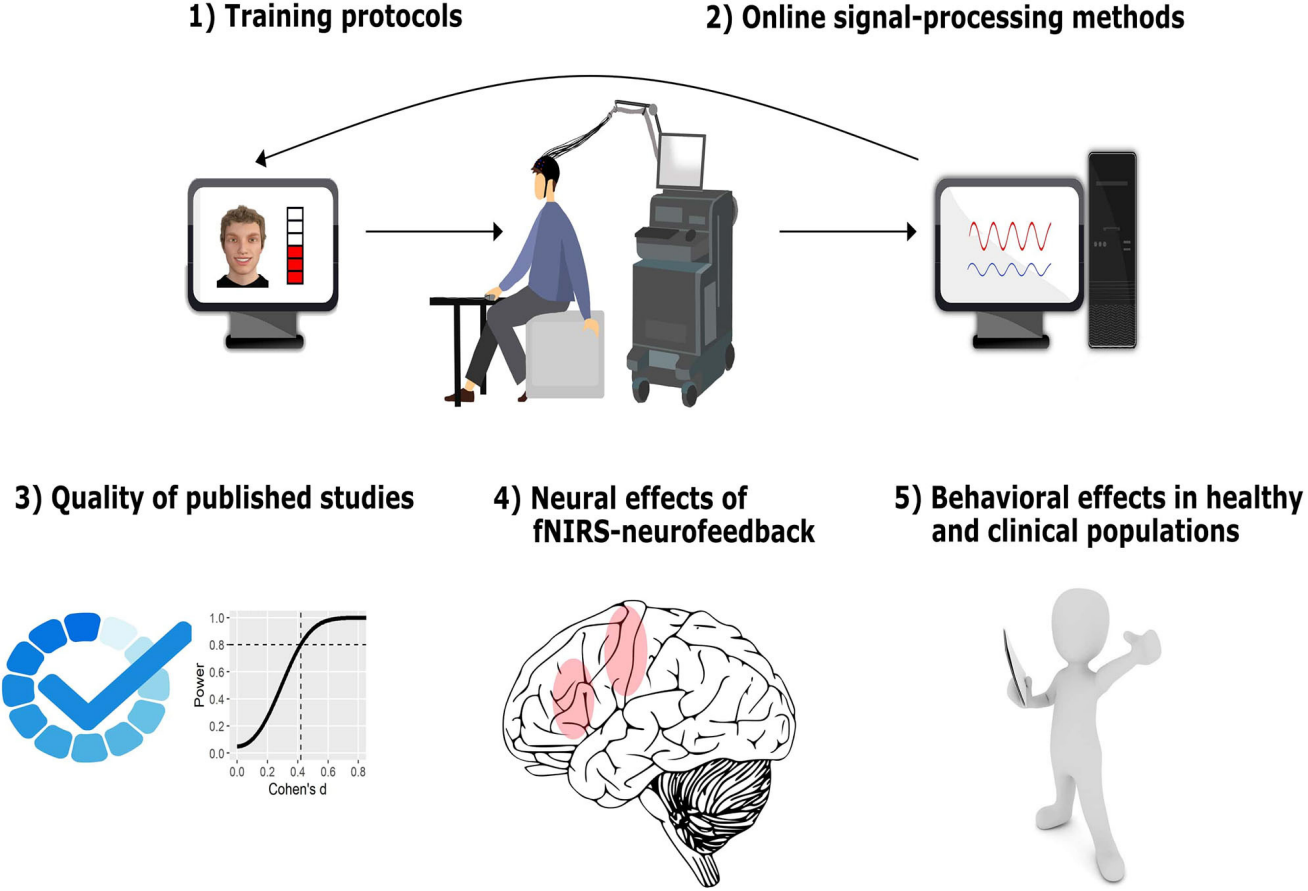
Structure of results and discussion. In the first two sections, we provide a comprehensive overview of how fNIRS-neurofeedback training is implemented, describing and discussing important features of neurofeedback-training protocols (1) and of the real-time signal-processing methods applied (2). In the third section, we critically evaluate the quality of published studies including experimental design and reporting quality as well as statistical power/sensitivity as an indicator of reliability of the reported findings (3). In the fourth section, we assess and discuss the effectiveness of fNIRS-neurofeedback to regulate and induce pre-post changes in brain activity (4). Finally, we assess and discuss its effectiveness in changing behavioral outcomes and we review the clinical potential of fNIRS-neurofeedback (5). The fNIRS illustration was created by Laura Bell.

We present and discuss the results of this systematic review in five sections (depicted in Figure 2).

### 1. Training Protocols

When designing a neurofeedback study and creating a new training protocol, there are a number of aspects to be considered. Some of these include: (1) population(s) to be studied (e.g., healthy or patients or both), (2) neural target for the neurofeedback training (e.g., a particular [set of] brain region(s) or a measure of connectivity), (3) control-group approach (see section 3.1), (4) duration of training, and (5) the neurofeedback procedure, including training conditions (e.g., regulation, rest, transfer), the kind of feedback presentation and task instructions. In this section of the review, we provide an overview of how previous fNIRS-neurofeedback studies established these protocol aspects. We also discuss implications and possible extensions for future studies. Table 1 shows details of the training protocols used in the studies included in our review.

**Table 1:** Neurofeedback training protocol details.

**Study**	**Data for section 1.2 and Figure 3B**	**Data for section 1.3**	**Data for section 1.4**			
	**Target**	**Training**	**Neurofeedback run periods and timing**	**Feedback**	**Instructed strategies**	**Regulation (total time)**
Aranyi et al. (2016)	Bilateral dlPFC asymmetry	1 session/1 practice run, 8 real runs/1 trial, 1 day of training	15 s rest/3 s instruction/40 s mental counting baseline/10 s rest/3 s instruction/40 s regulation/7 s rest	Immediate visual feedback, engagement (e.g., gaze, smile) of virtual agent	Express positive feelings toward the agent in order to capture its interest	6 min
Barth et al. (2016)	PFC	8 sessions/1 run/12 trials/ 30 s/8 days over 2 weeks	30 s relaxation (deactivation)/30 s regulation (activation)	Immediate visual feedback of all channels (color-coded)	Not reported	48 min
Fujimoto et al. (2017)	SMA	1 session real and 1 session sham NF/16 trials/5 s/2 days	8–15 s rest/5 s regulation	Immediate visual feedback, height and color of vertical bar	No strategies instructed	80 s
Hosseini et al. (2016)	dlPFC	4 sessions NF during verbal working memory task/1 run/80 trials/4 days over 2 weeks	8–10 s working memory trial (encoding and retention)/2 s test/6–8 s rest and feedback presentation	Delayed feedback, line plot displaying changes during previous ten trials	Meta-cognitive strategies	48 min
Hudak et al. (2017)	Bilateral dlPFC/IFG	8 sessions/2 runs NF/12 trials/1 run transfer/8 trials/session 1–4: 50/50%, session 5–8: 80/20% activation/deactivation trials/8 days over 2 weeks	20 s rest (5 s baseline)/30 s regulation/2 s reward	Immediate visual feedback, virtual classroom scenario: brightness of the lighting in the classroom, reinforcement after each trial	No strategies instructed	96 + 32 min transfer
Hudak et al. (2018)	Bilateral dlPFC/IFG	30 sessions/2 runs NF/12 trials/1 run transfer/8 trials/50/50% activation/deactivation trials/30 days over 12–49 weeks, 3 weeks intermission after session 15	30 rest (5 s baseline)/30 s regulation/2 s reward	Immediate visual feedback was presented via commercial EEG-NF system (moving objects and 2 s reinforcement)	Not reported	360 + 120 min transfer
Kanoh et al. (2011)	Left sensorimotor cortex	5 sessions/6 runs/5 trials/ 20 s/5 days of training	40–43 s rest/20 s up-regulation	Immediate visual feedback (length of white bar)	Motor imagery of right hand	50 min
Kimmig et al. (2018)	Bilateral dlPFC/IFG	15 sessions/2 runs NF/12 trials/1 run transfer/8 trials/75/25% up-/downregulation/15 days over 5–9 weeks, from 7th session: distractor background pictures with fear-related contents	30 s rest (5 s baseline)/30 s regulation/2 s reward	Immediate visual feedback, moving dot, and reinforcement “Well done!,” anxiety-related or neutral background pictures from 7th session onwards	Not reported	180 + 60 min transfer
Kinoshita et al. (2016)	Bilateral frontal pole cortex	1 session/6 runs (2 runs real/2 runs sham NF/2 runs transfer) 18 trials/1 day of training	16 s rest/10 s up-regulation	Immediate visual feedback (blue bar)	Memory, executive functions, and verbal fluency strategies suggested	6 min real + 6 min transfer
Kober et al. (2014)	Motor cortex asymmetry	8 sessions/2 runs/40 trials/8 different days	7–11 s rest/6–8 s regulation	Immediate visual feedback, moving dot + numerical score continuously updated	Kinesthetic motor imagery	~75 min
Kober et al. (2015)	Bilateral IFG	7 sessions/1 run/25 trials/7 different days	27–33 s rest/17–23 s up-regulation	Immediate visual feedback, moving dot + numerical score continuously updated	Motor imagery	~58 min
Kober et al. (2018)	Bilateral IFG	1 session/1 run/20 trials/1 day (also NF during rest, but instructed to relax and bring signal back to baseline)	30 s rest/17–23 s regulation	Immediate visual feedback (color-coded on a schematic head model)	Kinesthetic motor imagery	~7 min
Lapborisuth et al. (2017)	Left motor cortex	1 session/8 runs (motor imagery and execution)/4 runs with NF/4 runs without/6 trials	15 s rest/15 s up-regulation	Immediate visual feedback of all channels on a color-coded topographic image	Motor imagery/motor execution	6 min
Lee et al. (2015)	Sensory motor cortex	1 session, 2 runs, 1 run treadmill walking without NF, 1 run with NF	15 s rest/10 s up-regulation	Immediate visual feedback (red bar)	Not reported	100 s
Li et al. (2019)	Right lateral OFC	1 session, 1 run 6–10 trials to learn strategy, 4 real training runs/4 trials/one day/10–30 min break in between	25 s rest/25 s up-regulation	Immediate visual feedback, animation: “Lift a stone in front of a beach landscape”	No specific strategies instructed	~9–11 min
Liu et al. (2016)	Frontal and temporal face processing regions	5 sessions/2 runs/1 run: functional localizer/4 trials morphing faces/1 run training/10 trials, 5 days over 5 weeks	30 s rest/house-matching 20 s/face-matching (up-regulation) 28 s/2s feedback/reward display	Delayed feedback, points displayed after each trial, points were later converted to cash	No specific strategies instructed	~23 min
Marx et al. (2015)	Bilateral dlPFC/IFG	12 sessions/2 runs NF/12 trials/1 run transfer/8 trials, 50/50% activation/deactivation trials, 12 days within 4–6 weeks	25 s rest (5 s baseline)/30 s regulation/2 s reward	Immediate visual feedback was presented via commercial EEG-NF system (moving objects and 2 s reinforcement)	Not reported	144 + 48 min transfer
Mihara et al. (2012)	Left premotor cortex	1 session real/1 session sham NF/15 trials/1 day	8–15 s rest/5 s regulation	Immediate visual feedback, height and color of vertical bar	Kinesthetic motor imagery	75 s
Mihara et al. (2013)	Ipsilesional premotor cortex	6 sessions/32 trials/6 days over 2 weeks/10 min motor imagery training without NF before each session	8–15 s rest/5 s regulation	Immediate visual feedback, height and color of vertical bar	Kinesthetic motor imagery	16 min
Narita (2015)	Left PFC	2 sessions/7 runs/6 trials/2 days over 1 week? (but not clearly reported)	15 s rest/30 s regulation	Immediate visual feedback, color of monitor	Not reported	~42 min
Trambaiolli et al. (2018)	Frontal and occipital networks	1 session/2 runs classifier training/2 runs NF/11 trials (5) trials real, 3 trials fixed, 3 trials random feedback (neutral and positive affect conditions)	5 s fixation cross/2 s instruction/30 s positive or neutral affect condition/self-paced self-evaluation	Immediate visual feedback, amorphous figure	Imagine positive personal experiences	5 min
Weyand et al. (2015)	Bilateral PFC	16 sessions/3 runs/20–22 trials (up- and down-regulation)/15 sessions within 3 weeks, last session 10 days later/session 1–5: select strategy/session 6–10: practice strategies/session 11–15: stop strategies, use desire to regulate/session 16: follow-up	20 s rest/17 s regulation	Immediate visual feedback, color-coded topographic image, ball that rises and falls, and game feedback	Yes, specific strategies instructed	277 min

*dlPFC, dorsolateral prefrontal cortex; EEG, electroencephalography; IFG, inferior frontal gyrus; NF, neurofeedback; OFC, orbitofrontal cortex; PFC, prefrontal cortex; SMA, supplementary motor area*.

### 1.1. Target Populations

**Figure 3:**
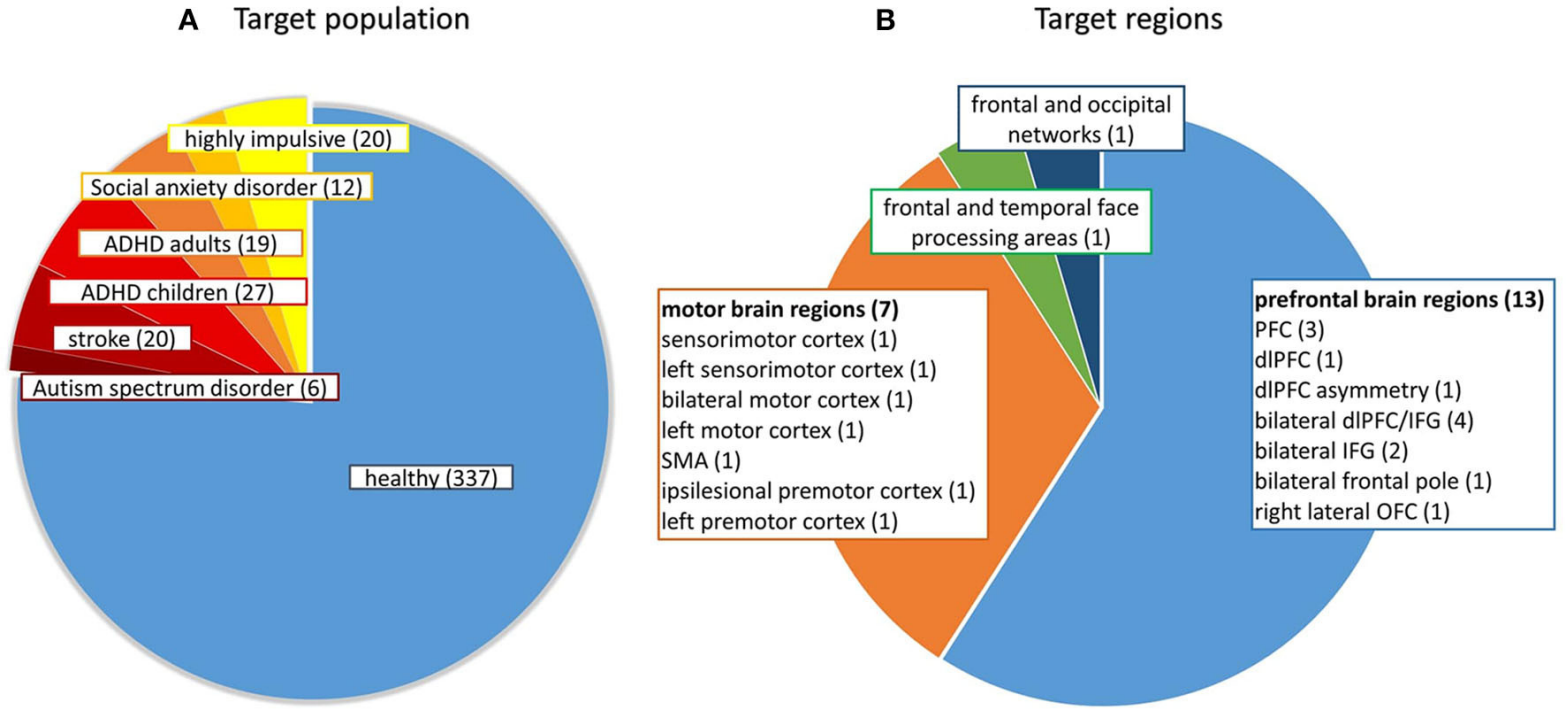
**(A)** Number of participants from different target populations and **(B)** Number of studies targeting a certain brain region. ADHD, attention-deficit/hyperactivity disorder; dlPFC, dorsolateral prefrontal cortex; IFG, inferior frontal gyrus; OFC, orbitofrontal cortex; PFC, prefrontal cortex; SMA, supplementary motor area.

Figure 3A shows the different target populations investigated in the studies. Mostly healthy participants were investigated (*N* = 337), but also patients after stroke (*N* = 20), with social anxiety disorder (*N* = 12), autism spectrum disorder (*N* = 6), attention-deficit/hyperactivity disorder (*N* = 27 children and *N* = 19 adults), and adults with high impulsivity (*N* = 20).

### 1.2. Target Regions

Figure 3B shows the distribution of the neurofeedback target regions of the included studies. The bulk of the studies trained participants to regulate parts of the prefrontal cortex (PFC), i.e., dorsolateral prefrontal cortex (dlPFC), inferior frontal gyrus (IFG), frontal pole or orbitofrontal cortex (OFC). Some studies broadly targeted the PFC using eight to fourteen target channels (Barth et al., 2016; Hudak et al., 2017, 2018; Kimmig et al., 2018) and others targeted specific subregions of the PFC using only one target channel (Li et al., 2019). Another large proportion of the studies trained participants to regulate activation within sensorimotor regions and enhance motor imagery-related brain activation (e.g., Fujimoto et al., 2017). One study reinforced up-regulation of temporal and frontal face-processing regions, as individually defined by a functional localizer (Liu et al., 2016) and another aimed to train a broad affective network of frontal and occipital brain regions using a multivariate classifier approach (Trambaiolli et al., 2018). The signal-to-noise (SNR) ratio likely differs between target regions and depends on scalp-brain or source-detector distance. The SNR is dependent on individual physical features such as individual brain anatomy, head size, skull thickness, and hair properties (e.g., thickness, density, length and color; Orihuela-Espina et al., 2010). On average, the scalp-brain distance is higher in parietal regions and lower in frontal and temporal regions (Cui et al., 2011). These factors should be taken into account when selecting target regions/channels for a neurofeedback study [see also section Selection of Target Regions (Channels of Interest)]. It should be noted that to date no study has used fNIRS-neurofeedback training to target connectivity between specific brain regions.

### 1.3. Training Duration

The length of training varied broadly between studies and ranged from short one-session designs (nine studies) up to 30 sessions (Hudak et al., 2018), resulting in a total duration of regulation training that varied between 75 s and up to 8 h. In particular, clinical treatment studies used a higher number of training sessions (Marx et al., 2015; Hudak et al., 2018; Kimmig et al., 2018). The majority of studies applied five sessions or less. While the optimal number of sessions for acquiring self-regulation of hemodynamic brain responses via fNIRS needs to be determined, successful regulation after even a single session has been reported in sham-controlled studies (Fujimoto et al., 2017; Li et al., 2019). Similar to fMRI-neurofeedback, fNIRS-neurofeedback targets spatially specific brain hemodynamics and might therefore offer a faster pace of learning compared to EEG-neurofeedback with most studies involving 20–40 training sessions (see also Marx et al., 2015; Thibault et al., 2018). However, the data of studies by (Marx et al., 2015; Mayer et al., 2015) that directly compared fNIRS- with EEG-neurofeedback still need to be published to shed further light on different learning mechanisms.

### 1.4. Neurofeedback Procedure

A neurofeedback procedure consists of at least three different aspects: (1) within-run task periods and their timing, (2) feedback presentation (i.e., sensory modality employed, timing, and complexity of feedback information, and (3) instructions provided to participants.

#### 1.4.1. Neurofeedback Run Periods and Their Timing

A neurofeedback run procedure consists of at least two different kinds of periods, i.e., a regulation period during which participants receive neurofeedback and try to change brain activity, and a resting period during which no feedback is provided that can also serve as a baseline-control condition. Most studies instructed participants to rest during the control condition, except for one study that instructed participants to engage in mental counting in an attempt to control for potentially confounding mental processes (Aranyi et al., 2016). Some protocols also included additional reward periods (e.g., smiling faces or points) presented after each regulation trial (Liu et al., 2016; Hudak et al., 2017, 2018; Kimmig et al., 2018), which is a form of additional delayed feedback. Two studies applied a combination of neurofeedback with (socio-)cognitive training and presented delayed feedback on activity during a cognitive task after a block (Hosseini et al., 2016; Liu et al., 2016). To ensure or assess the transfer of self-regulation skills beyond the neurofeedback sessions, a few studies employed transfer (no-feedback) periods (Marx et al., 2015; Hudak et al., 2017, 2018; Kimmig et al., 2018), where participants received the same instructions as in the neurofeedback task, but without receiving any feedback on their brain activity. Unfortunately, these studies did not report regulation success specifically for transfer periods. While most protocols either trained up- or downregulation, some also trained regulation in both directions, where activation and deactivation periods were randomly presented. The lengths of the regulation and baseline conditions varied broadly across studies from 5–40 to 6–43 s, with most studies varying between 20 and 30 s.

Transfer indicates that a skill is transferred to different situations or tasks. Depending on the contextual factors of the transfer situation, we can distinguish between *near* and *far transfer*, with the latter being more important for the success of training (see Barnett and Ceci, 2002). However, some studies (Marx et al., 2015; Hudak et al., 2017, 2018; Kimmig et al., 2018) used the term *transfer* to describe task periods but presented a reward after each *transfer* trial. Hence, they switched from immediate to delayed neurofeedback training and are unable to ensure or demonstrate that participants are able to regulate brain activity without receiving feedback, i.e., to transfer the skill of brain regulation to a new situation and possibly beyond neurofeedback training (*far transfer*). When combining up- and down-regulation within a session, care should be taken to balance the randomization of up- and down-regulation periods in order to keep transition probabilities between periods equal. Otherwise, participants may anticipate and prepare for the following condition, i.e., regulate in the opposing direction during the baseline condition as demonstrated by Hudak et al. (2018). Similarly, anticipatory effects might be prevented by introducing variable onsets of the regulation conditions, as applied by some of the studies. This would render randomization of periods unnecessary and enable to present several up- and downregulation periods in a blocked fashion, which reduces cognitive demands and may facilitate shaping of individual strategies during the training. However, combining up- and downregulation in a single session might involve the risk of carry-over effects between periods, which may impede regulation performance. However, this speculation needs to be corroborated by a comparative study. The optimal trial structure for neurofeedback procedures needs further investigation. Since comparative studies including fMRI- and EEG-neurofeedback research are lacking, we mostly rely on theoretical considerations when designing neurofeedback tasks. Considering the time course of the hemodynamic response (Ogawa et al., 1992), which is delayed and peaks after 4–6 s, a reasonable duration of the regulation and baseline period is 20–30 s. Further research is needed to discover whether shorter or longer periods of up to 40 s (Aranyi et al., 2016) and above are beneficial. While a short duration of 5 s can be considered too short for a hemodynamic response to develop properly, these studies continue to show the feedback signal during a subsequent rest period (Mihara et al., 2012, 2013; Fujimoto et al., 2017). Moreover, we can assume that if feedback is presented immediately (a slowly developing hemodynamic signal), longer durations are required compared to delayed feedback, which is presented considering the activation of a whole previous trial.

#### 1.4.2. Feedback Presentation

The presentation of feedback information may differ with regard to (1) sensory modality (visual, auditory, tactile), (2) timing (immediate vs. delayed), (3) complexity (simple vs. complex virtual environment), and (4) rewarding content (smiling faces or monetary reward). Most of the studies used a simple type of immediate visual feedback in the form of a bar (e.g., Fujimoto et al., 2017) or more complex animations of, for example, rising objects (e.g., Marx et al., 2015; Li et al., 2019), or smiling virtual agents (Aranyi et al., 2016). Others used color-coded topographic maps (Barth et al., 2016; Lapborisuth et al., 2017; Kober et al., 2018), displaying the signal change of all channels while instructing participants to focus on a certain region within the channel arrangement. Kimmig et al. (2018) started with a simple form of visual feedback (moving ball) and introduced neutral and anxiety-related pictures in the middle of the training period to provide a relevant context for patients with social anxiety disorder. Some studies presented delayed feedback in addition to immediate feedback in the form of a reward (e.g., smileys or points) after each trial (Hudak et al., 2017; Kimmig et al., 2018), or only delayed feedback in order to reduce distraction during the task/regulation period (Hosseini et al., 2016; Liu et al., 2016).

Whether immediate or delayed feedback is superior or whether they are equally effective is a matter of ongoing debate and there is only limited and as yet unclear evidence from a few comparative fMRI-neurofeedback studies (see Paret et al., 2019). In case of the noisier fNIRS-neurofeedback, feeding back mean or median activity at the end of each block may avoid confusing participants due to noisy fluctuations of the feedback signal and may also be beneficial with regard to timing (see above). The form of social neurofeedback used by Aranyi et al. (2016) may be experienced as more rewarding and may improve motivation, which is confirmed by preliminary evidence from an fMRI-neurofeedback study demonstrating that social neurofeedback outperforms simple visual feedback and leads to stronger activation of reward-related brain regions (Mathiak et al., 2015). In an effort to increase motivation and facilitate transfer to daily life or critical situations, neurofeedback has been embedded in a 3D virtual-reality environment. While the feasibility of this approach has been demonstrated in a subclinical adult population (Hudak et al., 2017), an ongoing clinical trial in children with attention-deficit/hyperactivity disorder (ADHD) is investigating whether virtual-reality-based neurofeedback is superior to simpler forms of feedback (Blume et al., 2017). Some researchers are opposed to complex feedback, arguing that neurofeedback should lead to knowledge of results and that gaming environments which are too complex may distract participants (Hinterberger et al., 2004; Sherlin et al., 2011). However, comparative studies provide evidence that the more engaging complex feedback embedded in a virtual environment may facilitate learning and is better received by users (Gruzelier et al., 2010; Cohen et al., 2016).

#### 1.4.3. Instructions

In a neurofeedback study, participants are provided with general task instructions about the experiment and in most cases also instructions on how to regulate a certain brain region. Instructions can be very specific (e.g., use motor imagery to regulate motor brain regions) or rather loose (e.g., use mental strategies to change the feedback signal).

Twelve studies instructed participants to upregulate, one to downregulate, and six to up- and down-regulate a certain target region. Another study reinforced asymmetry, i.e., a difference between the activity of a target region and its homolog on the other hemisphere, and two used a multivariate analysis approach to calculate the feedback signal. While some studies provided explicit instructions about types of mental strategies to regulate brain activity, such as kinesthetic motor imagery (Kober et al., 2014) or affective strategies (Trambaiolli et al., 2018), others merely encouraged participants to learn self-regulation using a trial-and-error approach (e.g., Fujimoto et al., 2017; Hudak et al., 2017).

Even if no strategies are provided, study design and general instructions about the experiment may prime the use of certain strategies (Kohl et al., 2019). For example, in the study by Fujimoto et al. (2017), when participants were informed about the target region (supplementary motor area) they might have been tempted to use motor-imagery strategies. Therefore, care needs to be taken when giving task instructions and also when informing participants about the general purpose of the experiment. It is thus important that participants' strategies are documented thoroughly, ideally after each neurofeedback trial or run [see also section Experimental Design and Reporting Quality (CRED-nf Checklist)], although results need to be interpreted carefully, since retrospective self-reports may lack realiability (see Veenman, 2011), particularly in children (Stone and Lemanek, 1990). Whether explicit instructions are beneficial for neurofeedback learning or not remains an open question and may depend on the particular training protocol (see Paret et al., 2019). For instance, it has been suggested that explicit strategies are not necessary, and some work even indicated that they may be detrimental or at least not helpful in some cases (Birbaumer et al., 2013; Sepulveda et al., 2016; Shibata et al., 2019). This is also supported by a recent fNIRS-neurofeedback study (Weyand et al., 2015) where participants used two personalized mental strategies over ten sessions and were then instructed to stop using their strategies and instead to use only their desire to regulate brain activity. Interestingly, regulation performance remained the same after weaning off specific mental strategies and the majority of participants reported training to be less demanding and more intuitive. Nevertheless, strategy instruction may initially facilitate learning (Scharnowski et al., 2015) and may be helpful if successful strategies to regulate a certain brain region are clearly known, e.g., using motor imagery to regulate motor regions. Hence, the decision about instructions may depend on the targeted brain region, duration, and purpose of the neurofeedback-training experiment.

### 1.5. Conclusion—Training Protocols

FNIRS-neurofeedback has been applied in a variety of populations, including different patient populations, children, adolescents, and older adults. However, previous studies are heterogeneous in terms of (i) selected neurofeedback targets (mostly comprising prefrontal and sensory motor brain regions), (ii) duration of training, and (iii) design of the neurofeedback procedure, including timing, feedback display, and instructions. Generally, as fNIRS measures the same hemodynamic brain signal as fMRI, a large part of the fMRI-neurofeedback training procedures can be (and have already been) transferred to fNIRS-neurofeedback. Note, however, that also for neurofeedback in general there are a lot of open issues with regard to training protocol methods (see Paret et al., 2019). Further systematic research and discussion will help to achieve consensus and make neurofeedback training protocols more efficient.

## 2. Online Signal-Processing Methods and Hardware

In this section, we give an overview of the different methods used for online signal-processing, including devices, selection of brain regions (fNIRS channels) of interest, online feature extraction (chromophores used), and preprocessing and artifact control (see also Table 2). Providing valid feedback of neural activity to the participant is a crucial component of successful neurofeedback applications. Therefore, methods have to be carefully selected to capture the neural activity of a target region and minimize strong extracranial artifacts, as are frequently present in the fNIRS signal (Caldwell et al., 2016).

**Table 2:** Online signal-processing methods and hardware.

**Study**	**Data for section 2.1**	**Data for section 2.2**	**Data for section 2.4**		**Data for section 2.3**		**Data for section 2.5**
	**Device**	**Selections of target regions [channel(s) of interest]**	**Online feature**	**Chromophore**	**Online preprocessing**	**Artifact control**	**Calculation of feedback signal**
Aranyi et al. (2016)	fNIR400	8 channels positioned on subject's forehead	Asymmetry (left vs. right dlPFC amplitudes)	HbO	Low-pass filter (finite impulse response, order 20) 0.1 Hz, sliding-window motion artifact rejection, reference channel	Reference channels	Threshold of signal based on mean, SD, and signal variation of signal during counting (baseline)
Barth et al. (2016)	ETG-4000	14 channels aligned with positions of EEG 10–20 system, registered to MNI space (Tsuzuki et al., 2007)	Amplitude, PFC	HbO	Not reported	None	Change in HbO compared to 15 s baseline at beginning.
Fujimoto et al. (2017)	OMM-3000	4 channels aligned with positions of EEG 10–20 system. MNI positions estimated using individual structural MRI and digitizer measurements	Amplitude (*t*-values estimated by GLM), SMA	HbO	GLM analysis. 20 s sliding window. Linear term to correct drift. 4 short-distance channels (principal component included as regressor in GLM).	EMG, 4 short-distance channels	Contrast regulation vs. rest (adaptive GLM, 20 s sliding window), maximum *t*-value from the 4 channels. Primary principal component of short-distance channels as nuisance regressor
Hosseini et al. (2016)	ETG-4000	Channels aligned with positions of EEG 10–20 system (Okamoto et al., 2004). Functional localizer (working memory task)	Amplitude, dlPFC	HbO	Bandpass filter 0.01–0.5 Hz	No	Change in the average HbO signal over feedback channels (over 9 s window) relative to the calibration period
Hudak et al. (2017)	ETG-4000	8 channels aligned with positions of EEG 10–20 system, registered to MNI space (Tsuzuki et al., 2007)	Amplitude, dlPFC/IFG	HbO	Kalman filter with a 5 s sliding window, CAR of all channels	CAR	Change in HbO compared to 5 s baseline, CAR of all channels subtracted
Hudak et al. (2018)	ETG-4000	8 channels aligned with positions of EEG 10–20 system, registered to MNI space (Tsuzuki et al., 2007)	Amplitude, dlPFC/IFG	HbO	Bandpass filter: 0.01–0.1 Hz, 5 s moving average, CAR of all channels	CAR	Change in HbO compared to 5 s baseline, CAR of all channels subtracted
Kanoh et al. (2011)	ETG-4000	3 channels aligned with positions of EEG 10–20 system	Amplitude sensorimotor cortex	HbO	High-pass filter and 7-point moving average	None	Average of channels/no baseline before task (not explicitly reported)
Kimmig et al. (2018)	ETG-4000	10 channels aligned with positions of EEG 10–20 system, registered to MNI space (Tsuzuki and Dan, 2014)	Amplitude, dlPFC/IFG	HbO	5 s moving average filter, CAR of remaining channels	CAR	Average of channels, change in HbO compared to 5 s baseline. Standard deviation from previous trial used to scale maximum/minimum of feedback signal. CAR of remaining channels subtracted.
Kinoshita et al. (2016)	ETG-4000	6 channels aligned with positions of EEG 10–20 system, registered to MNI space (Tsuzuki et al., 2007).	Amplitude, frontopolar cortex	HbO	Not reported	Respiratory rate	Average of channels, moving baseline, last 10 s. Maximum display of the bar graph was +0.25 [mMmm]
Kober et al. (2014)	ETG-4000	8 channels aligned with positions of EEG 10–20 system registered to MNI space (Singh et al., 2005). Functional localizer to select channels with best signal quality during a motor task	Asymmetry (difference between left and right motor area)	HbO	0.01 HPF and 1.5 Hz LPF, 2 s moving average. Difference of right and left channels (also cancels out artifacts)	4 reference channels	HbO left vs. HbO right. No baseline period before trials
Kober et al. (2015)	ETG-4000	4 channels aligned with positions of EEG 10–20 system, positions assessed via individual digitizer measurements	Amplitude (difference between IFG and posterior regions)	HbO and HbR group	0.01 high- and 1.5 Hz low-pass filter, 2 s moving average. Difference of IFG and of posterior reference channels	EMG, 4 reference channels	HbO/HbR of FB channels vs. reference channels. No baseline period before trials
Kober et al. (2018)	NIRSport	4 channels, positions assessed via individual digitizer measurements, probably based on EEG 10–20 system	Amplitude, IFG	HbO and HbR group	Not reported	None	Not reported
Lapborisuth et al. (2017)	LABNIRS	14 channels aligned with positions of EEG 10–20 system, positions assessed via individual digitizer measurements	Amplitude, motor cortex	HbO	Detrending and normalization (last 10 s)	None	Difference of current sample divided by SD of previous 10 s and linear trend of previous 10 s
Lee et al. (2015)	FOIRE-3000	7 channels aligned with positions of EEG 10–20 system. Positions assessed via individual digitizer measurements	Amplitude, sensorimotor cortex	HbO	Not reported	None	*t*-values, probably GLM
Li et al. (2019)	NIRSport	1 channel aligned with positions of EEG 10–20 system. Position of channel validated by MRI scans in two independent participants	Amplitude, OFC	HbO	2 s moving average	None	Change in HbO compared to 2 s baseline, feedback scaled based on a pre-experiment (“difficulty coefficient”)
Liu et al. (2016)	ETG-4000	Functional localizer at beginning of each session (channel with highest/lowest signal during face processing), positions assessed via individual digitizer measurements	Amplitude, frontal and temporal face processing regions	HbO	Reference channel (channel irrelevant for face-processing network identified during functional localizer)	Reference channel	HbO Percent signal change in feedback channel compared to reference channel (face matching compared to house matching)
Marx et al. (2015)	ETG-4000	8 channels aligned with positions of EEG 10–20 system, registered to MNI space (Tsuzuki et al., 2007)	Amplitude, dlPFC/IFG	HbO	Common average reference (CAR)	No	Change in HbO compared to 5 s baseline
Mihara et al. (2012)	OMM-3000	3 channels aligned with positions of EEG 10–20 system, positions estimated using structural MRI and digitizer measurements of representative participants	Amplitude (*t*-values estimated by GLM), premotor cortex	HbO	GLM analysis. 20 s sliding window, linear term to correct for drift. Autoregressive model order 1 to adjust autocorrelation, excluded 3 participants with finger movement	No, but reported offline control	Contrast regulation vs. rest (adaptive GLM, 20 s sliding window), maximum *t*-value from the 3 channels
Mihara et al. (2013)	OMM-3000	3 channels aligned with positions of EEG 10–20 system, positions estimated using individual MRI and digitizer measurements	Amplitude (*t*-values estimated by GLM), premotor cortex	HbO	GLM analysis, 20 s sliding window. Linear term to correct for drift, autoregressive model order 1 to adjust autocorrelation, EMG control	EMG	Contrast regulation vs. rest (adaptive GLM, 20 s sliding window), maximum *t*-value from the 3 channels
Narita (2015)	PocketNIRS, Dynasense	1 channel aligned with positions of EEG 10–20 system. No information about registration reported.	Amplitude, PFC	HbO	Not reported	None	Not reported
Trambaiolli et al. (2018)	NIRScout	32 channels aligned with positions of EEG 10–20 system. No information about registration reported	Output of classifier, frontal/occipital networks	HbO, HbR	1 Hz low-pass filter and 3 s moving average, normalization by average signal from the same channel during previous neutral condition	None	Output of linear discriminant classifier based on HbO and HbR of 32 channels, indicating neutral or positive affect
Weyand et al. (2015)	Imagent	9 channels aligned with positions of EEG 10–20 system. No information about registration reported	Classifier, PFC	HbO, HbR, tHb	Third-order Chebyshev IIR cascade filter with pass-band edge frequency of 0.1 Hz, stop-band edge frequency of 0.5 Hz, and pass band ripple of 0.1 dB	None	Session 1–5: weighted slope score. Session 6–16: output of classifier. LDA classifier using 8 features selected from 288 different temporal and spatiotemporal features from all channels and chromophores

*CAR, common average reference; COI, channel(s) of interest; dlPFC, dorsolateral prefrontal cortex; EEG, electroencephalography; EMG, electromyography; IFG, inferior frontal gyrus; HbR, deoxyhemoglobin; LDA, linear discriminant analysis; HbO, oxyhemoglobin; PFC, prefrontal cortex; tHb total hemoglobin*.

### 2.1. Devices

Nine different devices were used in the studies: ETG-4000, OMM-3000, NIRSport, NIRScout, fNIR400, LABNIRS, FOIRE-3000, PocketNIRS, and Imagent. Some devices were used more frequently than others. However, this does not imply qualitative superiority, and it is beyond the scope of this review to judge the functionality of the devices used. Notably, other commercially available devices are used in cognitive neuroscience and custom-developed mobile fNIRS instrumentations (also available open-source: www.opennirs.org) are used for BCI applications (von Lühmann et al., 2015), and may be used for neurofeedback applications. For an overview of commercially available fNIRS systems and their features refer to Scholkmann et al. (2014) and Pinti et al. (2018a). Major differences between the devices are the wavelength used and the amount of wavelength exploited by the systems. Five of the devices used two wavelengths (ETG-4000, NIRSport, NIRScout, Imagent, fNIR400) whereas the other four devices support three wavelengths (OMM-3000, FOIRE-3000, LABNIRS, PocketNIRS). It should be noted that each of the devices used different wavelengths in the range from 690 to 860 nm and had different precisions (e.g., dynamic range or sensitivity, based on the hardware and its quality). These differences can potentially constitute confounds in the context of reproducibility of neurofeedback results. The use of more wavelengths can improve signal quality (Arifler et al., 2015) and might therefore be beneficial for fNIRS-neurofeedback applications.

### 2.2. Selection of Target Regions (Channels of Interest)

In order to target specific brain regions reliably, the studies applied different methods to verify appropriate channel selection for extracting the neurofeedback information. In fNIRS-neurofeedback studies, a certain number of optodes are placed on the participants' heads (general optode setup), then some of the channels (‘channels of interest') are selected to extract the neurofeedback information. All the studies used *a priori* knowledge about the assumed location of specific brain regions involved in the regulation task. Additionally, most studies used the EEG 10–20 system (Jasper, 1958) as a reference to place the optodes (20 out of 22 studies; Table 2) and the methods of Okamoto et al. (2004), Singh et al. (2005), or Tsuzuki et al. (2007) to register channels to the MNI space. Alternatively (or in combination), the positions were either verified by an individual or reference structural MRI scan combined with a digitizer measurement (Fujimoto et al., 2017) or a functional localization procedure was performed. Eight studies used additional digitizer measurements to verify channel positions *post*-*hoc*. Three studies used a functional localizer before the training session to select task-relevant channel(s) with individually good signal quality (Kober et al., 2014; Hosseini et al., 2016; Liu et al., 2016). In one study, the optodes were placed on the forehead of the subject without describing the use of other reference points (Aranyi et al., 2016). Three studies provided no further information about additional registration of the optodes. For the eight studies using digitizer measurements, the channels were positioned with respect to the neurofeedback target region based on MNI coordinates using virtual spatial registration or general head location in combination with a functional localizer (Liu et al., 2016). The input for the online feature was either a single channel or the average signal of channels covering the region of interest. The number of channels used for extracting the neurofeedback information ranged from 1 to 14 for studies using amplitude changes and 9 to 32 channels for studies using multivariate statistics as a feature for the feedback (see Table 2).

In sum, the selection of channels of interest relied mainly on the EEG 10–20 system in combination with MNI coordinates and *a priori* knowledge about specific brain regions that are involved in the regulation task, which is a suitable procedure for selecting target channels. A combination with functional localizers seems to be a more reliable solution (if applicable) for amplitude-based studies since it additionally takes subject-specific variance into account by selecting individualized channels. Additionally, using digitization of optode locations and alignment with (preferably individual) MRI scans allows even more details to be obtained on optimum placement of the optodes.

### 2.3. Online Pre-processing and Artifact Control

The fNIRS signal comprises different sources of noise. Most problematic in the context of real-time analysis seems to be the physiological noise that overlaps partly with task frequency, such as low-frequency oscillations of blood pressure (Mayer waves; Kamran et al., 2016). The studies applied different online preprocessing methods to deconfound the feedback signal from these sources of noise. Five studies did not report the online preprocessing methods they used and are thus not further considered in this review with respect to their preprocessing (Lee et al., 2015; Narita, 2015; Barth et al., 2016; Kinoshita et al., 2016; Kober et al., 2018). The majority of the studies which reported their online pre-processing steps applied different kinds of high- and low-pass filters (10 of 17; Table 2). Some studies did not use a high-pass filter (e.g., Aranyi et al., 2016; Kimmig et al., 2018; Li et al., 2019). For low-pass filtering, mainly finite impulse response (FIR) filters were used, most commonly a moving-average filter (window ranging from 2 to 5 s). Four studies (Marx et al., 2015; Hudak et al., 2017, 2018; Kimmig et al., 2018) used a common average reference (CAR) for data preprocessing, i.e., subtracting the average of all channels from the feedback channel, three of these in combination with additional filtering. Four studies used one or multiple reference channels (Kober et al., 2014, 2015; Aranyi et al., 2016; Liu et al., 2016). However, considering the lack of relevant information in other studies, it may be the case that some of these studies used additional filters but did not report them. Most of the studies did not use any explicit artifact control on top of the filtering methods (7 of 17). The studies that used artifact control to some degree most frequently employed either CAR or reference channels (8 out of 11) and one study (Kober et al., 2015) also included electromyography (EMG) measures for *post*-*ho*c artifact control. Only one of these eight studies used short-distance channels to control for artifacts (in combination with EMG measures, Fujimoto et al., 2017). Two studies used either only EMG (Mihara et al., 2013) or the respiration rate (Kinoshita et al., 2016) as a reference for *post*-*ho*c artifact control, which was implemented using, for example, visual inspection of the EMG signal or differences in the respiration rate. Aranyi et al. (2016) used a sliding-window motion artifact rejection (SMAR) procedure, which rejected motion-affected periods in the fNIRS signal. Other motion correction methods suitable for real-time applications, e.g., Cui et al. (2010), were not applied.

Surprisingly, only three out of the nine studies that targeted motor regions used EMG to control for subtle movements (Mihara et al., 2013; Kober et al., 2015; Fujimoto et al., 2017), which can confound the feedback signal, and details of the EMG analysis are rarely reported. Other studies did not control for this confound or only visually inspected movements of the participants (Mihara et al., 2012). It is important that future studies targeting motor regions control for this confound. To date, studies have only looked at motion artifacts *post*-*hoc*. Future studies could establish methods to control for EMG signals online, e.g., stop presenting feedback signal when movements occur or include the EMG signal as a nuisance regressor when using a general linear model (GLM) approach to calculate the feedback signal. Some studies did not apply high-pass filtering. It could be argued that if the feedback signal is compared to a preceding baseline in a short time frame, low-frequency drifts can be neglected, which would render high-pass filtering unnecessary. However, this remains to be confirmed by future research.

In general, the field would benefit from implementing more sophisticated artifact-control methods to account for potential confounding signals (Caldwell et al., 2016; Tachtsidis and Scholkmann, 2016; Pfeifer et al., 2018). Short-distance channels in combination with GLM seem to be the most efficient tool to correct for extracerebral physiological signal components (Brigadoi and Cooper, 2015; Tachtsidis and Scholkmann, 2016; von Lühmann et al., 2020). As already stated, only Fujimoto et al. (2017) used this technique, which may be because most of the fNIRS systems are not equipped with the appropriate hardware (Klein and Kranczioch, 2019). If this is the case, a potential alternative is the global component removal approach as introduced by Zhang et al. (2016). This technique seems to be promising to reduce global physiological signals from the fNIRS data and can be used for online artifact control as recently pointed out by Klein and Kranczioch (2019) specifically with respect to single-trial data. FNIRS-neurofeedback studies have not yet applied this method but have rather used CAR or other referencing to correct for evoked systemic cerebral and extracerebral components. However, referencing should be applied with care, as reference channels have to be independent of the target region and participants may modulate the feedback signal by regulating reference channels instead of feedback channels (Hudak et al., 2018). Therefore, the global component-removal approach could be a viable alternative. However, if none of those methods is available during the experiment it should be checked *post*-*hoc* that these global signals did not drive the neurofeedback signal change.

### 2.4. Online Feature—Chromophores Used

For the online feature, either the amplitude or a derivative of the HbO or HbR was used, or a classifier was trained to discriminate specific states. Most studies (20 of 22) used a type of amplitude change. In examining the studies that used the amplitude, we found three studies (Mihara et al., 2012, 2013; Fujimoto et al., 2017) which used the maximum *t*-value of the selected channels. All other studies used the HbO or HbR amplitude as a direct source for the feedback, scaled to a certain level, and referenced to a specific period before the feedback. For the studies using a classifier, a linear discriminant analysis (LDA) classifier was trained to discriminate two classes (e.g., neutral or positive affect; Weyand et al., 2015; Trambaiolli et al., 2018).

Even though there are multiple options for the source of neurofeedback information using fNIRS, most of the studies used HbO. Only two of the 22 studies used both chromophores and only two used HbR and HbO for different study groups (Kober et al., 2015, 2018). It is important to note that two of the four studies that used both chromophores employed a classifier approach (Trambaiolli et al., 2018), and one also included tHb (Weyand et al., 2015). All other studies used the direct amplitude of HbO/HbR or a derivative of it. Kober et al. (2018) showed that people can regulate both chromophores with fNIRS-neurofeedback, but depending on the regulation strategy (in this case motor imagery to alter brain activation) regulation ability may be restricted to the natural course of the HbO and HbR signal changes related to this strategy.

The best-suited chromophore for neurofeedback (and other BCI) applications is still a matter of debate and has not been intensively investigated. Most studies use HbO since it displays larger amplitudes than HbR (Stangl et al., 2013; Sato et al., 2016). On the other hand, Kirilina et al. (2012) reported HbR to be less sensitive to artifacts. However, a recent study (Klein and Kranczioch, 2019) demonstrated that HbR is also affected by a global signal component. The contrast-to-noise ratio seems to be comparable for HbO and HbR across different tasks (Cui et al., 2011), but according to Naseer and Hong (2015) HbO signals were more discriminative for BCI applications than those of HbR signals.

In sum, although less frequently used, current evidence does not imply that HbR or tHb are less suitable for fNIRS-neurofeedback. While all three options (HbO, HbR and tHb) seem to be suitable, future research is still needed to ascertain whether one option outperforms the others in the context of neurofeedback applications.

### 2.5. Calculation of Feedback Information

The calculation of the feedback signal greatly depends on the type of display used for the presentation (see section 1.4.2). Generally, the amplitude of the HbO signal during the feedback/task block was used and either compared to a preceding baseline (e.g., Hudak et al., 2017, 2018), the fNIRS-system baseline (Barth et al., 2016), or a baseline of the GLM (e.g., Fujimoto et al., 2017). The signal of interest can additionally be compared to a different channel not covering the region of interest (Liu et al., 2016) or asymmetry scores can be calculated, e.g., difference between right- and left-hemispheric channels (Aranyi et al., 2016). For the two studies using a multivariate approach, the feedback was based on the output of the classifier identifying neutral or positive affect (Weyand et al., 2015; Trambaiolli et al., 2018). Two studies (Narita, 2015; Kober et al., 2018) did not report the methods used for feedback calculation. Using the system baseline instead of a preceding baseline before each trial seems risky, since low-frequency drifts may confound the signal if the applied preprocessing methods do not capture them properly.

Another important aspect is the selection of feedback thresholds, i.e., defining the amount of signal change necessary for change and setting a minimum and maximum of the presented feedback. For example, thresholds were defined based on signal variation during the preceding control condition (Aranyi et al., 2016; Kimmig et al., 2018) on the basis of *t*-values, which was also used for feedback (Fujimoto et al., 2017), or based on a certain HbO change (Kinoshita et al., 2016). Li et al. (2019) calibrated thresholds based on a pre-experiment conducted in an independent sample. However, some studies did not transparently report how the threshold used for feedback was defined [see also section 3.2].

### 2.6. Conclusion—Online Signal-Processing Methods and Hardware

While the studies reviewed applied a considerable diversity of online signal-processing methods, some similarities across the studies were also evident, e.g., regarding online-feature and chromophore selection. Unfortunately, crucial information regarding online signal-processing procedures was often missing. Since there are no established standards for online or offline processing methods (Kamran et al., 2016; Pinti et al., 2018b), future studies are encouraged to explore different methods and provide sufficient information so that other studies can easily replicate successful methods.

Assuring signal quality is crucial for neurofeedback applications, particularly for fNIRS-neurofeedback, which suffers from strong extracranial artifacts. A careful selection of online signal-processing methods is necessary to avoid the presentation of invalid feedback information. Further developments and more systematic research on fNIRS online signal-processing methods are definitely needed to overcome this specific shortcoming of the fNIRS technology.

## 3. QUALITY OF PUBLISHED STUDIES

In this section, we assess and discuss the quality of published studies including (1) features of experimental designs and methodological quality according to the JBI ratings, (2) design and reporting quality according to the CRED-nf checklist, and (3) analysis of statistical power/sensitivity as an indicator of reliability of the reported findings.

### 3.1. Quality of Experimental Designs

Table 3 shows important features of the experimental designs of the studies.

**Table 3:** Study designs.

**Study**	**Data for 1.2 and Figure 3B**	**Data for 1.2 and Figure 3A**	**Data for 3.1**				
	**Target**	**Participants**	**Control group**	**Randomization**	**Blinding**	**Follow up**	**Transfer**
Aranyi et al. (2016)	dlPFC asymmetry	18 healthy	None	No	No	No	No
Barth et al. (2016)	PFC	13 healthy	None	No	No	No	Separate task
Fujimoto et al. (2017)	SMA	20 healthy	Sham (yoked feedback, within)	Yes	Single-blinded	No	No
Hosseini et al. (2016)	dlPFC	20 healthy	Sham (yoked feedback)	No	Not reported	No	No
Hudak et al. (2017)	Bilateral dlPFC/IFG	20 highly impulsive	EMG biofeedback	Yes	No	No	Separate tasks
Hudak et al. (2018)—excerpt from Mayer et al. (2015)	bilateral dlPFC/IFG	19 adults with ADHD	None	No	No	Not reported, but protocol included FU	No
Kanoh et al. (2011)	Left sensorimotor cortex	5 healthy	None	No	No	No	Separate task
Kimmig et al. (2018)	Bilateral dlPFC/IFG	12 SAD	None	No	No	No	Separate task
Kinoshita et al. (2016)	Bilateral frontal pole cortex	24 healthy	Sham feedback (artificially generated, within)	Yes	Single-blinded	No	Yes
Kober et al. (2014)	Motor cortex asymmetry	17 healthy	Sham (yoked feedback, within)	Yes	Single-blinded	No	No
Kober et al. (2015)	Bilateral IFG	20 healthy	HbO vs. HbR-group	Yes	Single-blinded	No	Separate task
Kober et al. (2018)	Bilateral IFG	48 healthy/12 per group	Bidirectional control for HbO and HbR	Yes	Single-blinded	No	No
Lapborisuth et al. (2017)	Left motor cortex	22 healthy	Motor imagery without feedback (within)	No	No	No	Yes
Lee et al. (2015)	Sensory motor cortex (S1, M1, SMA)	4 healthy	Motor task without feedback (within)	Unclear	No	No	No
Li et al. (2019)	Right lateral OFC	60 healthy	Sham (yoked feedback)	Yes	Single-blinded	No	No
Liu et al. (2016)	Frontal and temporal face processing regions	2 healthy, 2 ASD	Sham feedback (artificially generated)	Yes	Not reported	No	No
Marx et al. (2015)	Bilateral dlPFC/IFG	27 children ADHD/9 per group	EEG and EMG biofeedback	No	No	2 weeks and 6 months	Separate tasks
Mihara et al. (2012)	Left premotor cortex	21 healthy	Sham feedback (artificially generated, within)	Yes	Single-blinded	No	No
Mihara et al. (2013)	Ipsilesional premotor cortex	20 stroke patients	Sham feedback (artificially generated)	Yes	Double-blinded	2 weeks	No
Narita (2015)	Left PFC	4 ASD	None	No	No	1–3 months	No
Trambaiolli et al. (2018)	Frontal and occipital networks	33 healthy	Sham feedback (artificially generated, within)	Conditions presented in random order	Single-blinded? Not clearly reported	No	No
Weyand et al. (2015)	Bilateral PFC	10 healthy	None	No	No	10 days	No

*ADHD, attention-deficit/hyperactivity disorder; ASD, autism spectrum disorder; ADHD dlPFC, dorsolateral prefrontal cortex; EEG, electroencephalography; EMG, electromyography; IFG, inferior frontal gyrus; HbR, deoxyhemoglobin; OFC, orbitofrontal cortex; HbO, oxyhemoglobin; PFC, prefrontal cortex; SAD, social anxiety disorder; SMA, supplementary motor area*.

#### 3.1.1. Control Conditions

Depending on the specific research aim, neurofeedback studies can make use of several different control conditions. Control conditions may include, treatment-as-usual, bidirectional-regulation control, feedback of an alternative brain signal, sham feedback, and mental-rehearsal control, and can be applied in a within- or between-subject design (see Sorger et al., 2019). Ideally, multiple control conditions are applied in order to disentangle neurofeedback-specific from unspecific processes. In this regard, Lubianiker et al. (2019) recently proposed an extension of established control conditions, in which participants of the control group are randomly assigned to a subset of different neural control targets (randomized ROI control condition). In this way, specific effects related to the control targets and neurofeedback-unspecific processes that likely differ for different neurofeedback targets may average out across all subjects of this control group. For extensive discussions on different control conditions in neurofeedback research, see Lubianiker et al. (2019) and Sorger et al. (2019).

Seven of the studies did not use any control condition. Nine studies used a sham feedback based on either artificially created signals (five studies) or based on a brain signal of another participant (yoked feedback, four studies). Two studies compared the effects with other forms of biofeedback (EEG- or EMG-based). One study compared the effects of motor-imagery neurofeedback with mental rehearsal (motor imagery only) and another compared neurofeedback during a motor task with motor task only in a within-subject design. Also, the effects of neurofeedback based on HbO and HbR were compared in a between-subject design without an additional sham-feedback condition (Kober et al., 2015) and in a bidirectional-control approach, investigating four different groups (Kober et al., 2018).

#### 3.1.2. Randomization and Blinding

Of the fifteen studies with a control group, eleven studies randomized assignment to groups or order of conditions. Seven studies blinded participants to conditions and only one applied double-blinding. However, in some situations blinding is not possible, e.g., when comparing neurofeedback to another treatment. These studies attempted to reduce bias by not informing participants about any other treatment than that which they received (Marx et al., 2015; Hudak et al., 2017), which may at least help to keep levels of expectation and motivation equal across groups. Assessments of motivation, expectation, or other unspecific factors can be included in future studies to check this assumption.

#### 3.1.3. Assessment of Transfer

To assess a transfer effect, four studies made use of transfer trials or no-feedback conditions, where participants received the same instructions as in the neurofeedback task, but without receiving any neurofeedback information. As described in section 1.4, some studies used a very liberal definition of the term transfer (if at all a very *near transfer*; see Barnett and Ceci, 2002). Five studies (Mihara et al., 2012; Kober et al., 2015; Barth et al., 2016; Hudak et al., 2017; Kimmig et al., 2018) assessed transfer using other computerized tasks and investigated whether activation within the targeted brain region changes after neurofeedback training, and were able to demonstrate transfer beyond neurofeedback training (far transfer).

#### 3.1.4. Follow-Up Measures

Only four of the studies investigated long-term effects of neurofeedback in a follow-up (10 days up to 6 months after neurofeedback training) in order to investigate whether participants were still able to regulate brain activity after a period without training (Weyand et al., 2015) or stability of observed behavioral effects (Marx et al., 2015). Evidence of delayed effects of neurofeedback emerging after the primary endpoint of a study has been reported. Particularly when investigating clinical populations, follow-up measures may boost statistical power and should be applied where possible (Rance et al., 2018; Van Doren et al., 2019).

#### 3.1.5. Methodological Quality (JBI Critical Appraisal Tool)

To assess the methodological quality of the included studies, we used the checklist for quasi-experimental studies of the Joanna Briggs Institute (JBI) critical appraisal tools (Tufanaru et al., 2017). Two of the authors (SK and DM) independently rated studies according to the nine criteria of the checklist. These items include: *clarity of cause and effect (temporal relationship between variables), similar participants; similar treatment in compared groups; existence of a control group/condition; multiple measurement points of the outcome; completion of follow-up; similar outcome measurements in compared groups; reliability of outcome measurements; appropriate statistical methods*. Each study was allocated points based on the number of criteria fulfilled. Disagreements between the review authors were resolved by discussion. For further details about rating criteria, see Supplementary Material.

Table S1 shows the results of the ratings for each study. On average 5.55 (*SD* = 2.15) of 9 required quality criteria were rated “yes.” It should be noted that only four studies used appropriate statistical methods according to this rating. Most of the studies were rated as not using appropriate statistical methods because they did not justify their sampling plan or omitted labeling their study a *pilot, feasibility* or *proof-of-concept* study.

### 3.2. Experimental Design and Reporting Quality (CRED-nf Checklist)

To assess experimental design and reporting quality we used the current version of the CRED-nf (Consensus on the reporting and experimental design of clinical and cognitive-behavioral neurofeedback studies) checklist (Ros et al., 2020). The CRED-nf checklist is designed to encourage best practice in terms of experimental designs and reporting of neurofeedback studies. It covers seven domains (*Pre-experiment, Control groups, Control measures, Feedback specifications, Outcome measures brain, Outcome measures behavior, and Data storage*), including 23 checklist items, fifteen of which are considered *essential*, and eight *encouraged*. In contrast to the JBI checklist, the CRED-nf checklist does not include subjective ratings. Instead, it assesses whether a study reports contents required by a respective item, e.g., “Report the feedback modality and content.” One of the authors (SK) filled in a checklist for each study with page numbers identifying where each point was addressed. The number of addressed items of both categories *essential* and *encouraged* are reported for each study. For further details see Supplementary Material.

Table 4 and Table S2 show detailed results of the CRED-nf checklist, including a short description of individual items of the respective CRED-nf domains. On average 63.03% (~9 of 15) of the essential items and 10.23% (~1 of 8) of encouraged items were reported. It should be noted that this best-practice checklist was published only very recently after all the included studies had been published. Hence, the authors of the included studies as well as ongoing studies could not make use of this resource in designing their experiments and publishing their results.

**Table 4:** Reporting and design quality according to the CRED-nf checklist.

**CRED-nf domain**	**M ± SD (%)**
Pre-experiment	20.45 ± 25.16
Control groups	25.45 ± 22.41
Control measures	42.73 ± 22.51
Feedback specifications	90.00 ± 13.45
Outcome measures—brain	53.03 ± 31.97
Outcome measures—behavior	18.18 ± 24.62
Data storage	0.00 ± 0.00
CRED-nf essential	63.03 ± 18.49
CRED-nf encouraged	10.23 ± 9.94
CRED-nf total	44.66 ± 13.56

*Mean percentages and standard deviations of items rated “yes” for the different domains of the CRED-nf checklist. Detailed results for individual studies and items, including a description of the items of the respective domains, can be found in Table S2. CRED-nf, consensus on the reporting and experimental design of clinical and cognitive-behavioral neurofeedback studies checklist (Ros et al., 2020)*.

The domain *Pre-experiment* was not very well reported, on average only 20.45% of the items were included. Only one study reported a pre-registration of the experimental protocol (Hudak et al., 2018). However, this study deviated from the originally published registration (Mayer et al., 2015) and investigated a different research question including only a subsample of participants. None of the studies conducted a power analysis in order to justify sample size. Eight of the 22 studies were labeled as a *pilot, feasibility*, or *proof-of-concept* study, which renders a power analysis unnecessary.

On average only 25.45% of the items in the domain *Control groups* were reported, which is related to the fact that only a few clinical trials have yet been published and seven uncontrolled studies were included. Of the fifteen controlled studies, nine reported having used single- or double-blinding or at least discussed the fact that blinding was not possible (Marx et al., 2015; Hudak et al., 2017). Only one study reported having blinded raters of the outcomes or whether participants/experimenters remained blinded. Two of the clinical studies employed a standard-of-care intervention group as a benchmark for improvement (Mihara et al., 2013; Marx et al., 2015).

With respect to the domain *Control measures*, the studies reported on average 42.73% of the items. Six studies reported having used some measure of psychosocial or non-specific factors (e.g., motivation, expectation, effort). Since these measures are easy to implement and do not require much additional time, we recommend that future studies should make more use of this additional easy and low-cost method of controlling for non-specific effects. Almost a third of the studies did not report whether participants were provided with a strategy and only six studies reported strategies used by the participants. Even if no explicit strategies are provided, study design and general instructions about the experiment may prime the use of certain strategies. The use of mental strategies undoubtedly affects brain activity and may also induce behavioral effects on its own. Hence, better control and transparent and more detailed reporting of this factor is required, which may also contribute to solving outstanding issues of the utility of strategy instructions (see also section Neurofeedback Run Periods and Their Timing).

The domain *Feedback specifications* was well reported, on average 90% were included. All studies included feedback modality and content as well as the software and hardware used, and almost all studies reported the definition of the online-feature extraction. Sixteen of the studies also included at least some information about the reinforcement schedule, for example feedback-threshold criteria, but did not justify this in relation to the existing literature. Also, the amount of reward received by participants was rarely included. While most studies reported the essential contrast used for feedback, i.e., regulation *vs*. rest, only eight of the studies described both conditions of regulation and rest separately.

Studies reported on average 53.03% of the items related to *Outcome measures—Brain*. Most studies included neurofeedback regulation success based on the feedback signal. However, some studies did not report regulation success at all (Marx et al., 2015; Narita, 2015), while others only reported regulation success of representative participants or only based on significant channels and took surrounding channels that were not used for feedback calculation into account instead of reporting the average of all feedback channels (e.g., Mihara et al., 2012; Lapborisuth et al., 2017). This lack of transparency makes it difficult to conclude whether neurofeedback was at all effective on the brain level. Less than half of the studies plotted the feedback signal of within-session or between-session regulation blocks of the feedback signal, which would permit further insights into the dynamics of regulation performance over the course of the training period. Only seven of the studies statistically compared the experimental to the control condition or group. There were seven uncontrolled studies unable to make such a comparison. Hence, seven controlled studies did not undertake this comparison, but rather analyzed groups/conditions separately, which does not allow any conclusions to be drawn about group differences and makes it difficult to appraise the effectiveness of neurofeedback (Nieuwenhuis et al., 2011).

On average only 18.18% of items in the domain *Outcome measures—Behavior* were reported. However, this low rate can be explained by the fact that most studies were mainly interested in regulation performance, and behavioral variables did not represent the primary outcome or were not assessed at all. Of the 14 studies that actually assessed a behavioral variable, eight reported a correlation between regulation success and behavioral outcome. None of the studies included measures of clinical or behavioral significance, such as the minimal clinically important difference (MCID; Engel et al., 2018). However, there are only a few clinical studies in which such a measure is applicable.

None of the studies reported having uploaded materials, analysis scripts, code, or data to an open-access data repository.

#### 3.2.1. CRED-nf Checklist—Conclusion

In sum, the reporting quality of published studies can be considered to be moderate to low in the light of current consensus guidelines. While some domains such as *Feedback specifications* were well reported, other such as *Pre-experiment* or *Data Storage* were not. The CRED-nf checklist provides guidelines for best practice with regard to experimental design and reporting of neurofeedback studies. The field of fNIRS-neurofeedback is still very young and some of the standards set by the CRED-nf checklist do not yet apply at this early stage. For example, double-blinding or a measure of clinical or behavioral significance should be included at the stage of randomized controlled trials, but not necessarily in early proof-of-concept studies that primarily aim to test neural regulation performance. However, we note that we applied more lenient criteria for some of the items, which resulted in higher ratings than if we had applied the original items. This should be considered in future comparisons applying the CRED-nf checklist. As stated above, this best-practice checklist was not available when the studies included in this review were published. Hence, a lack of reporting for some items should be expected at this early stage. Nevertheless, the incomplete reporting identified in the domain *Outcome measures—Brain* gives reason for concern. Without this information, we cannot clearly identify whether participants learned to control the brain signal of interest. As is common across the published literature, findings that do not meet a threshold for statistical significance may remain underreported in the fNIRS-neurofeedback literature. Future studies could benefit from orienting toward these new guidelines, while also abiding by other guidelines for reporting clinical trials, such as CONSORT (Schulz et al., 2010). We encourage researchers to use the CRED-nf checklist as an orientation when designing new studies and suggest they make use of the CRED-nf web application (rtfin.org/CREDnf) when submitting results for publication. The web application helps authors to standardize the reporting of CRED-nf items, which will facilitate future systematic reviews.

### 3.3. Statistical Power/Sensitivity

To further assess study quality, we extracted information about the reported sample sizes and estimated statistical power/sensitivity of the included studies. Statistical power, the probability of detecting an effect size of a certain magnitude with a given sample size and accepted threshold for the probability of a false-positive finding, is an important indicator for the reliability of reported findings and will therefore be investigated here.

The majority of neuroscientific studies, including neuroimaging studies, feature relatively small sample sizes and hence remarkably low statistical power for detecting realistic (i.e., small to moderate) effect sizes (Button et al., 2013; Poldrack et al., 2017). Furthermore, small sample sizes imply higher variability around effect size estimates. In combination with publication bias (i.e., the tendency to publish mainly significant findings), reported effects thus tend to be overestimated (Algermissen and Mehler, 2018), rendering the scientific literature in psychology and neuroscience an unreliable basis for conducting power analyses for future studies (Szucs and Ioannidis, 2017; Allen and Mehler, 2019; Schäfer and Schwarz, 2019).

Computing statistical power requires knowledge of the effect size that is considered relevant and worth detecting, i.e., the smallest effect size of interest (SESOI). *Post*-*hoc* power analyses that are commonly not based on SESOIs represent mere transformations of the *p*-value. Such an approach cannot therefore provide any information about the *a priori* power of the included fNIRS studies. Since SESOIs are highly experiment-specific (Lakens et al., 2018) and unknown to us, it proved difficult to set an SESOI for (1) regulation performance and (2) behavioral outcomes for our purpose. First, we note that neurofeedback experiments represent complex interventions (Sitaram et al., 2017) during which various factors, or their interaction, may determine the outcome (see also section 1.4). We also note that neurofeedback studies vary greatly in how they define and quantify successful regulation (Paret et al., 2019) and different definitions may reveal different effect sizes. Second, SESOIs for behavioral outcomes will depend on the specific paradigm (e.g., motor imagery or emotion regulation), outcome variable (e.g., reaction times or self-rated measures of emotion regulation), and study population (i.e., healthy participants or patient populations, and for patient groups the type of clinical population, e.g., stroke or depressed patients). Therefore, we did not set an SESOI to calculate the *post*-*hoc* statistical power of fNIRS-neurofeedback studies, but instead used Cohen's conventions, covering a range of effect sizes that may be comparable to potential SESOIs (*d* = 0.2, 0.5, and 0.8; Cohen, 1992).

Additionally, we assessed the statistical sensitivity, calculating the smallest effect size that individual studies were able to detect with certain probabilities (0.8 and 0.95), given their reported sample size. We conducted separate analyses for regulation performance and for a behavioral outcome, where applicable. Several studies employed a repeated-measure analysis of variance (ANOVA) but did not provide details of the correlation among repeated measures or violation of the sphericity assumption. For pragmatic reasons, we thus assumed for all studies that there was no violation of sphericity and a correlation of 0.8, which is considered a good general estimate for test-retest correlations in neuropsychological assessments (Calamia et al., 2013). To simplify analyses, we also used uncorrected *p*-values and ANOVA/*t*-tests instead of non-parametric tests, if used by a specific study. Additionally, instead of mixed models or ANOVAs with more than one within factor, two-factorial mixed ANOVAs were used for the sensitivity analysis. Overall, these measures should lead to higher power estimation, and we can assume that we overestimated the statistical power of the studies. Analyses were carried out using G^*^Power (Faul et al., 2007).

Overall, sample sizes varied across studies from our minimum inclusion size, i.e., four (two per cell), up to 60 (30 per cell). One single group study included 33 participants. The median sample size of the studies was 20 (12 per cell). We calculated the median effect size that was detectable with 80 and 95% power. For the outcome regulation performance, we found a value of *d* = 0.85 and *d* = 1.13, respectively. For behavioral outcomes, the value was *d* = 1.28 and *d* = 1.63, respectively. We further found that the median power to detect a small effect of *d* = 0.2 was low (0.14 and 0.08). Our results showed that the power was even insufficient to reliably detect large behavioral effects (0.43). Studies were only powered to reliably detect large effects in regulation performance (0.75; see Table 5 and Tables S3, S4 for more details).

**Table 5:** Sensitivity and statistical power for reported analysis.

**Study**	***N***	**Sensitivity**		**Power to detect**		
		**80% power**	**95% power**	***d* = 0.2**	***d* = 0.5**	***d* = 0.8**
**Regulation performance**						
Mean	19.29	*d* = 1.06	*d* = 1.38	0.14	0.41	0.66
Median	19	*d* = 0.85	*d* = 1.13	0.14	0.43	0.75
**Behavioral outcomes**						
Mean	22.1	*d* = 1.11	*d* = 1.45	0.10	0.31	0.56
Median	20	*d* = 1.30	*d* = 1.66	0.08	0.22	0.42

*Note that in order to simplify the analysis for some studies, we performed the analysis for a different statistical test than originally reported, did not take into account correction for multiple comparisons, and assumed no sphericity violation and a high correlation among repeated measures for ANOVAs. Overall, these measures should have led to an overestimation of the statistical power/sensitivity of the studies*.

Some studies that included control groups lacked a direct statistical comparison of regulation performance between the experimental and the control condition. Instead, these studies only reported the main effects within conditions and compared statistical significance between conditions instead of effect sizes. This statistical approach is erroneous for group comparisons (Nieuwenhuis et al., 2011) and makes it difficult to assess whether the experimental group outperformed the control group. If we assume that these studies found no statistically significant group effect, we note that due to insufficient statistical power we cannot come to valid conclusions about potential group effects. To check this assumption, we conducted sensitivity analyses for the respective group/interaction effects, using a statistical test that was appropriate for the respective study design (see Supplementary Material). Sensitivity for detecting a certain group/interaction effect size was lower as compared to detecting a within-group main effect. It is reasonable to assume that interaction effects are smaller and, depending on the underlying assumptions, require four to sixteen times more participants to achieve similar a priori statistical power (Gelman, 2018). Therefore, studies were likely underpowered for reliably detecting group differences in neurofeedback effects, which are very likely smaller than within-group effects.

As noted earlier, it remains difficult, if not impossible, to generalize these findings across paradigms because the smallest relevant effect sizes (SESOIs) may depend on the choice of the neurofeedback target region, population, control conditions, and other characteristics of the design. However, specific behavioral effects of neurofeedback, as assessed in placebo and motivation level-controlled designs, are possibly rather small (see Supplementary Material). This assumption is at least partly supported for the EEG-neurofeedback literature where well-controlled studies depending on population and rating report no specific group effect (e.g., Schabus et al., 2017; Schönenberg et al., 2017) or medium to small and non-significant effect sizes (Strehl et al., 2017).

**Figure 4:**
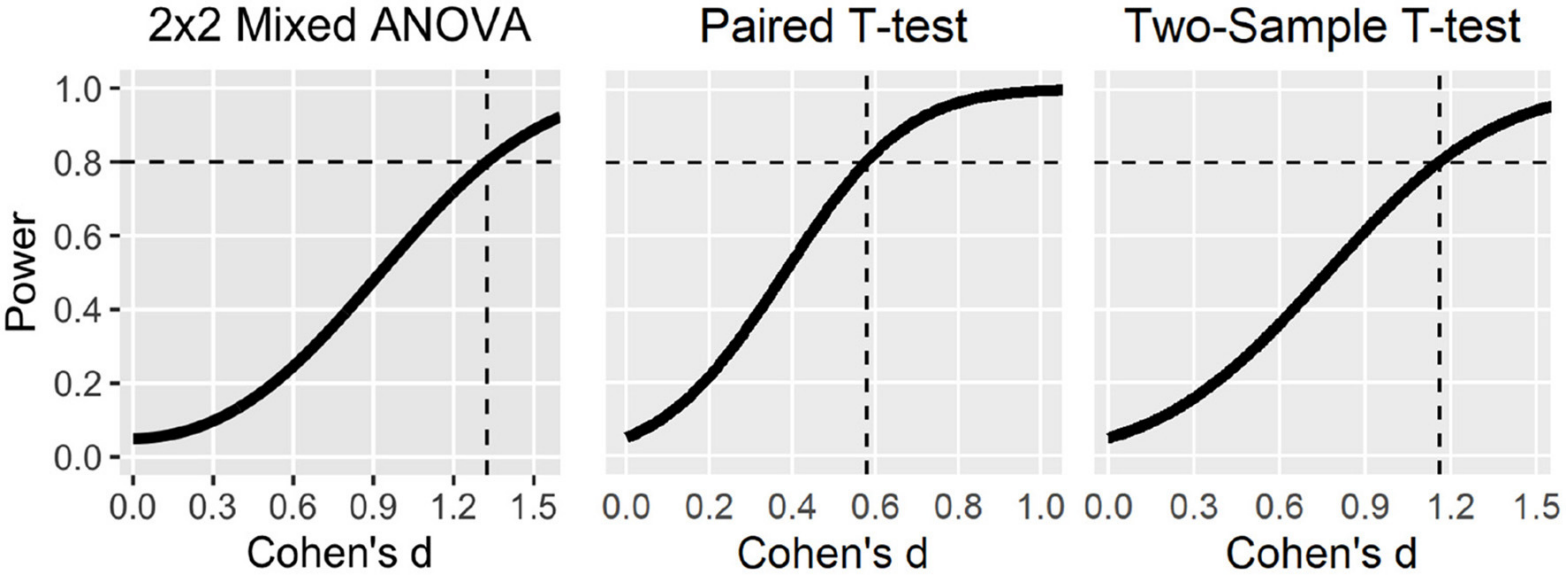
Statistical power curves to detect different effect sizes with 20 participants (median sample size) for different statistical tests. Dashed lines indicate smallest effect sizes detectable at 80% power. Note that the power curve for the 2 × 2 mixed ANOVA was based on liberal statistical assumptions (e.g., high correlation among repeated measures, sphericity, and uncorrected *p*-value of 0.05).

Altogether, our results suggest the median sample size of published fNIRS-neurofeedback studies is relatively small at *N* = 20, which is comparable to sample sizes reported more broadly for neuroimaging (Poldrack et al., 2017). It is thus not surprising that most studies lack sufficient statistical sensitivity to detect (realistic) SESOIs. In contrast, most studies were biased toward finding only relatively large effects (Figure 4). We further note that the analyses we present are based on rather liberal statistical assumptions (e.g., no application of multiple-testing correction) and thus likely still overestimate the true statistical sensitivity and power in the field.

Particularly with regard to specific behavioral effects, studies still lack statistical sensitivity and thus allow only very limited conclusions to be drawn about the feasibility of the paradigms and no conclusions about the specificity of fNIRS-neurofeedback effects. The field mainly consists of small feasibility, pilot, or proof-of-concept studies, which do not have to fulfill the same requirements with regard to sample size since their purpose is to explore the potential of fNIRS-neurofeedback in self-regulating a target brain signal and modulating behavior. However, as described above, most studies are not sufficiently transparent about this and hence risk overstating their findings. We thus reiterate previous concerns and recommend that authors appropriately label pilot, feasibility, and proof-of-concept studies, ideally in their manuscript title. Furthermore, we recommend a clear distinction between planned and exploratory analyses.

### 3.4. Conclusion—Quality of Published Studies

**Figure 5:**
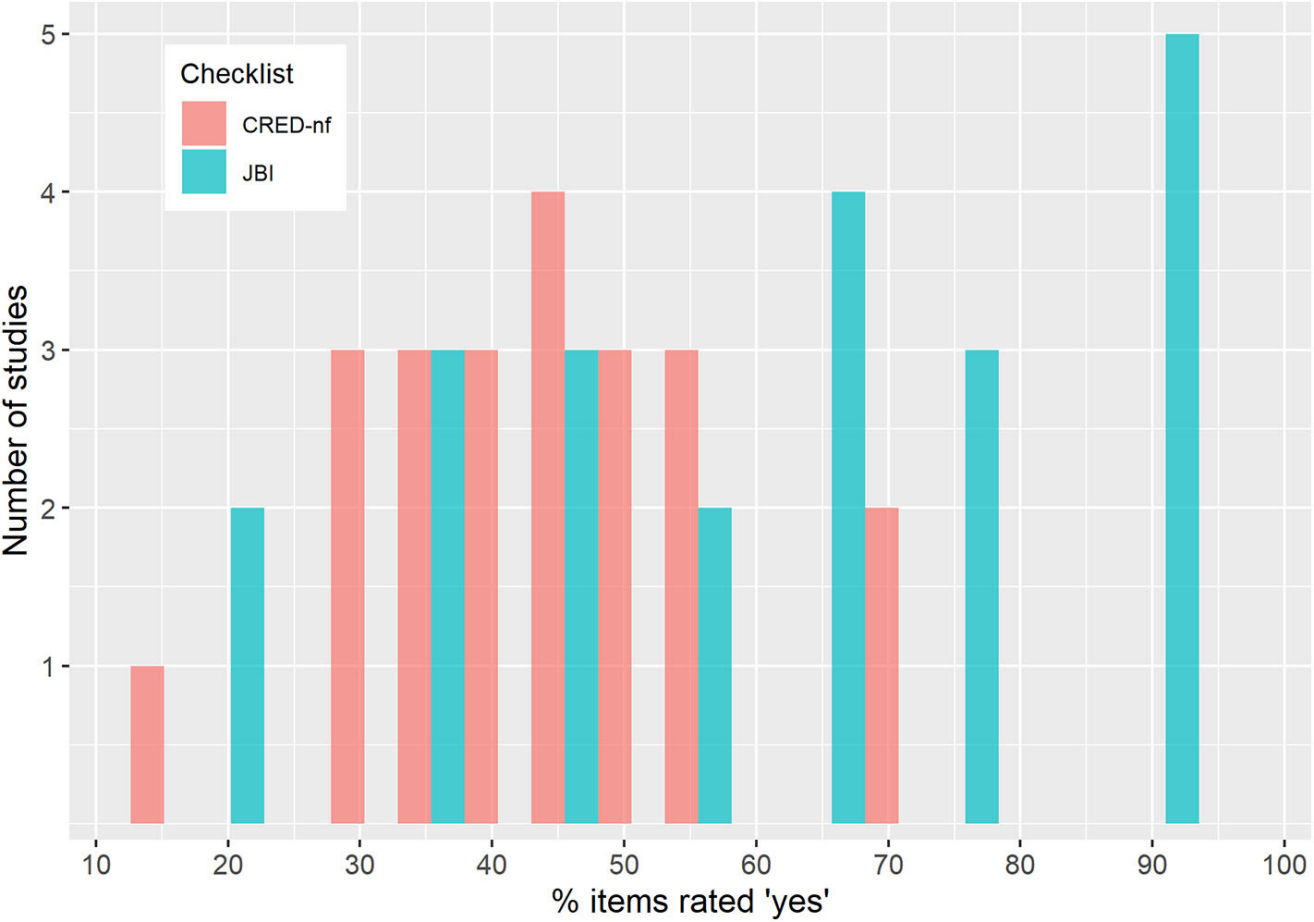
Quality of studies according to the CRED-nf and JBI checklist.

Altogether, the design and reporting quality of the studies can be considered to be moderate. There were a few studies of lower quality, but also some high-quality studies including sham-control conditions, randomization, and blinding (Figure 5). Sample sizes of the studies were small and thus their statistical power to detect realistic effects was low. While similar results have been reported for other fields within the neurosciences (Nieuwenhuis et al., 2011; Button et al., 2013; Szucs and Ioannidis, 2017), fNIRS-neurofeedback is still in its infancy and we thus still have the chance to tackle these issues early on and lay a more robust foundation to build upon in future work. As the field moves on, well-designed, sufficiently powered confirmatory studies are necessary to reach valid conclusions about the effectiveness of fNIRS-neurofeedback.

## 4. Neural Effects of Neurofeedback

In this section, we assess and discuss the effectiveness of fNIRS-neurofeedback for regulating and inducing pre-post changes in brain activity.

### 4.1. Neurofeedback Regulation Success

Before considering training effects in terms of changes in behavior or brain activity after neurofeedback, an essential question is whether neurofeedback training was successful, meaning was it effective for regulating the target brain signal. Unfortunately, no standard has been established so far, and there are a number of different ways to define and quantify neurofeedback regulation success. Paret et al. (2019) provide a taxonomy and discuss this issue in depth. In this section, we first discuss different success measures as applied by the studies and then systematically assess and critically discuss the effectiveness of fNIRS-neurofeedback for regulating a target brain signal, as reported by the studies (see Table 6).

**Table 6:** Neurofeedback regulation success.

**Study**	**Data for 1.2 and Figure 3B**	**Data for 1.2 and Figure 3A**	**Data for 4.1**	**Data for 4.1 and** **Figure 6**			
	**Target region**	**Target population**	**Success measure and analysis**	**Successful regulation as compared to**			
				**CTB**	**ECTL**	**Linear**	**CTC**
Aranyi et al. (2016)	dlPFC asymmetry	18 healthy	*Success rate*. Successful trial = statistically significant increase in average asymmetry during NF compared to counting baseline (*t*-test)	Yes	NR	No	N/A
Barth et al. (2016)	PFC	13 healthy	*Fixed threshold*. Average increase of HbO over whole training course	Yes	NR	NR	N/A
Fujimoto et al. (2017)	SMA	20 healthy	*Fixed threshold*. Significant increase in HbO during late compared to early trials (time/condition interaction)	Yes	Yes	NR	Yes
Hosseini et al. (2016)	dlPFC	20 healthy	*Fixed threshold*. Linear regression over trials within and over sessions. Further group analysis offline (but overlap with feedback channels unclear)	No	No	No^a^	NR^b^
Hudak et al. (2017)	Bilateral dlPFC/IFG	20 highly impulsive	*Success rate*. Successful trial = at least 7 s of the last 15 s regulation in the desired direction. Average of all trials from first four sessions compared to last four sessions	NR	No	No	NR
Hudak et al. (2018) -	Bilateral dlPFC/IFG	19 adults with ADHD	*Success rate*. Successful trial = at least 7 s of the last 15 s regulation in the desired direction. Average of all trials from first half compared to last half of sessions	Yes	No	No	N/A
Kanoh et al. (2011)	Left sensorimotor cortex	5 healthy	*Fixed threshold*. Linear regression over sessions	NR	NR	No	N/A
Kimmig et al. (2018)	Bilateral dlPFC/IFG	12 SAD	*Success rate*. Successful trial = at least 7 s of the last 15 s regulation in the desired direction. Last three sessions compared to first three sessions.	NR	Yes	NR	N/A
Kinoshita et al. (2016)	Bilateral frontal pole cortex	24 healthy	*Fixed threshold*. Increased HbO in feedback channels compared to rest baseline	Yes	NR	NR	No
Kober et al. (2014)	Motor cortex asymmetry	17 healthy	*Fixed threshold*. Last three sessions vs. first three sessions	Yes	Yes	Yes	Yes
Kober et al. (2015)	Bilateral IFG	20 healthy	*Fixed threshold*. HbR increased over sessions in HbR group and HbO decreased. In HbO group HbR increased over session as well.	Yes/No^c^	NR	Yes/No^c^	NR
Kober et al. (2018)	Bilateral IFG	48 healthy/12 per group	*Fixed threshold*. Not compared to baseline, motor imagery and no group comparison	NR	NR	Yes/No^c^	NR
Lapborisuth et al. (2017)	Left motor cortex	22 healthy	*Fixed threshold*. Not clearly reported. Defined ROI based on motor execution task (overlap with feedback channels not clear) and analyzed HbR instead of HbO. No comparison between conditions and no effect for HbO reported	NR	NR	NR	NR
Lee et al. (2015)	Sensory motor cortex	4 healthy	*Fixed threshold*. Increased HbO in feedback channels. No statistical comparison between feedback and no feedback condition.	NR	NR	NR	NR
Li et al. (2019)	Right lateral OFC	60 healthy	*Fixed threshold*. Significant increase in HbO over NF runs (time × group interaction). Regional specificity also confirmed by exploratory analysis of all channels	Yes	Yes	Yes	Yes
Liu et al. (2016)	Frontal and temporal face processing regions	2 healthy, 2 ASD	Not reported	NR	NR	NR	NR
Marx et al. (2015)	Bilateral dlPFC/IFG	27 children ADHD/9 per group	Not reported	NR	NR	NR	NR
Mihara et al. (2012)	Left premotor cortex	21 healthy	*Fixed threshold*. Increase in HbO compared to baseline for only one of the three feedback channels reported	Yes	NR	NR	No
Mihara et al. (2013)	Ipsilesional premotor cortex	20 stroke patients	*Fixed threshold*. Increased activation in one of the three FB channels compared to baseline. Timeline analysis and ROI analysis. Statistical details about timeline analysis missing	Yes	Yes	NR	Yes
Narita (2015)	Left PFC	4 ASD	Not reported	NR	NR	NR	N/A
Trambaiolli et al. (2018)	Frontal and occipital networks	33 healthy	*Success rate*. Successful trial = classifier. Percentage of successful trials for all conditions	Yes	NR	NR	No
Weyand et al. (2015)	Bilateral PFC	10 healthy	*Success rate*. Successful trial = classifier. Average classification accuracy for each session	Yes	NR	NR	N/A

*ADHD, attention-deficit/hyperactivity disorder; ASD, autism spectrum disorder; CTB, compared to baseline; ECTL, early compared to late, trail(s); CTC, compared to control group or within control condition; dlPFC, dorsolateral prefrontal cortex; IFG, inferior frontal gyrus; HbR, deoxyhemoglobin; NF, neurofeedback; OFC, orbitofrontal cortex; HbO, oxyhemoglobin; PFC, prefrontal cortex; SAD, social anxiety disorder; SMA, supplementary motor area*.

a *Marginal effects were considered “no,” only significant effects were considered “yes”*.

b *Effects were reported based on an offline analysis without clarifying whether significant effects overlapped with feedback channels and were therefore not considered in our analysis*.

c *Kober et al. (2015, 2018) trained regulation of HbO and HbR in separate groups*.

#### 4.1.1. Neurofeedback Regulation Success—Measures

While most fNIRS-neurofeedback studies define regulation success based on the magnitude of a signal change, some also define it on the basis of time, i.e., on the amount of time in a trial during which the feedback signal exceeds a defined threshold (Hudak et al., 2017, 2018; Kimmig et al., 2018). Most studies apply a *fixed threshold* approach and compare the regulation statistically to a baseline condition over a session, while some also report *success rates*, i.e., a ratio of successful (as previously defined) trial per session (Weyand et al., 2015; Aranyi et al., 2016; Trambaiolli et al., 2018) or for a group of sessions (Hudak et al., 2017, 2018; Kimmig et al., 2018). None of the studies calculated *personal effect sizes*, i.e., divided average signal change by the standard deviation of a session or run to account for individual noise (cf. Paret et al., 2019). Finally, to judge the regulation success of neurofeedback training, effects over time should be assessed for potential learning curves, rather than merely assessing average signal change across all trials compared to the baseline. For instance, comparisons can be made between early [first session(s)/trial(s)] and late parts of the training period [last sessions(s)/trial(s)] (Kober et al., 2014; Fujimoto et al., 2017; Kimmig et al., 2018). Alternatively, assuming linear improvement, regulation success can be assessed over all trials or sessions via linear regression (Kober et al., 2018; Li et al., 2019). Ultimately, success measures should be compared with a control group or within control conditions. This is important since also sham feedback induces activations, particularly in frontal brain regions (Ninaus et al., 2013), which is the target of the majority of fNIRS-neurofeedback studies. Certain studies compared some of the first sessions with some of the last sessions (Kober et al., 2014; Kimmig et al., 2018), which seems arbitrary. If certain sessions or trials are selected for comparison, this should be theoretically or empirically justified. For example Fujimoto et al. (2017) compared the first six trials with the last ten trials, because according to the existing literature participants should reach a plateau after the first six trials during a motor-learning task (Hatakenaka et al., 2007).

It should be noted that, with a few exceptions in fMRI-neurofeedback (e.g., Goldway et al., 2019), variability measures have been neglected in most neurofeedback success definitions. According to learning theories, the probability of a certain behavior (brain activity) should increase after learning (Skinner, 1963), but variability (noise) should also be reduced. Some theorists have compared neurofeedback learning to motor skill learning (Sitaram et al., 2017). From this perspective, it is assumed that in the course of learning a new skill variability is reduced to optimize performance (He et al., 2016).

#### 4.1.2. Neurofeedback Regulation Success—Results

**Figure 6:**
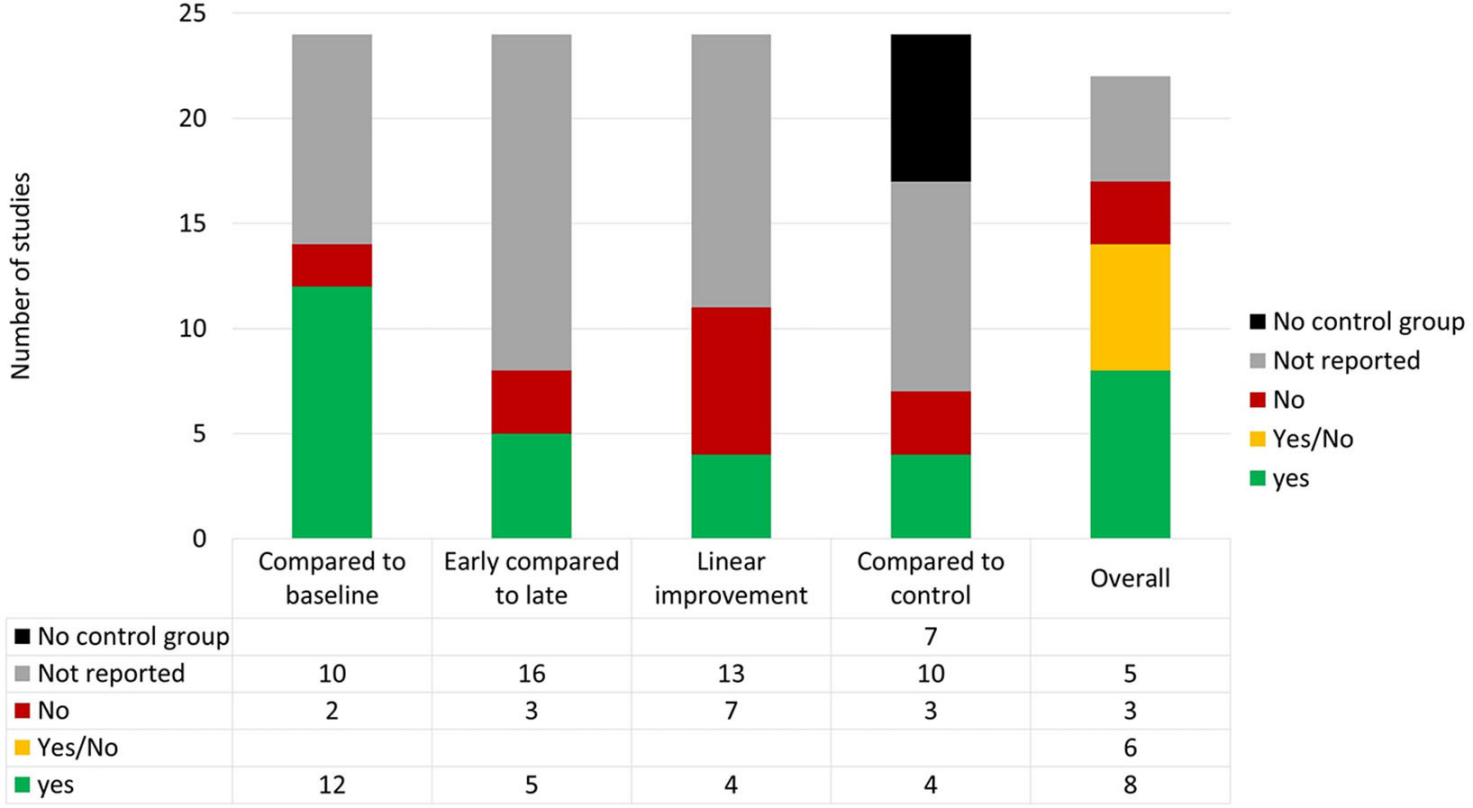
Neurofeedback regulation success. Overall regulation was classified “Yes” if a significant effect for one or more of the four measures were reported and “No” if no significant effect was reported. If both were reported, the overall regulation was classified “Yes/No.” Note that Kober et al. (2015, 2018) trained the regulation of HbO and HbR in different groups and found differential results for the groups. Therefore, the two studies were counted twice for the four measures. In overall regulation, the two studies were only counted once and were classified as “Yes/No”.

We assessed whether the studies reported an effect for each of the aforementioned comparisons (see Table 6 and Figure 6). Overall, results show that fNIRS-neurofeedback can be used to regulate brain activity, with some of the studies also demonstrating a greater increase over time as compared to a control group (Mihara et al., 2013; Kober et al., 2014; Fujimoto et al., 2017; Li et al., 2019). Particularly these studies were all of higher quality according to our ratings, at least single- or double-blinded and applied a sham-feedback approach (see section 3.1). The other studies reported mixed results, lacked a control group, or did not report results sufficiently well, making it difficult to draw definite conclusions. For example, some studies did not report regulation success based on the feedback signal, but rather results from an offline analysis of all channels, and did not clarify whether significant effects overlap with channels used for feedback (e.g., Mihara et al., 2012; Lapborisuth et al., 2017). This impedes any judgement about the success of a neurofeedback protocol. We encourage authors to report regulation success based on all feedback channels. If more than one channel is used, the average of the channels or an ROI analysis only based on feedback channels can be reported. Furthermore, some of the studies used time-based binary success criteria and did not additionally report brain activation during regulation. These criteria require the definition of a threshold to be surpassed and neglect information about the signal amplitude. Some studies (Hudak et al., 2017, 2018; Kimmig et al., 2018) used a threshold of zero, i.e., spent at least half of the time of the last 15 s of a trial in the desired direction. This threshold is very liberal and it can be expected that random fluctuation (only noise) of a signal should be half of the time above and half of the time below zero. Hence, high success rates can be expected by chance and are not informative. When using time-based criteria, the amplitudes of the feedback signal should be reported as well.

### 4.2. Neural Changes Over Time and Neural Mechanisms

In addition to analyzing regulation success, some studies explored pre-post changes in neural outcomes or investigated neural mechanisms of neurofeedback as assessed during training. Hudak et al. (2017) found increased activation of the left dlPFC (part of the region trained) during NoGo trials after neurofeedback compared to an active control group, but there was also a baseline difference between groups, and decreased activity in the experimental group before neurofeedback training may explain the effect. No effect was reported for the working-memory task, which is in line with (Barth et al., 2016), who reported only marginal decreases for frontal and language-related brain regions during a working-memory task. Kimmig et al. (2018) found no change in social-threat processing-related brain activity after neurofeedback, but a change in the right inferior parietal sulcus, right inferior frontal gyrus, and supplementary motor cortex correlated positively with a change in social anxiety. Mihara et al. (2012) found increased activation of the left premotor cortex in channels not used for feedback, and decreased activity in the parietal association cortex, which was related to an increase in the sham-feedback group, speculatively related to switching to visual imagery strategies due to the incorrect feedback in this group. Kober et al. (2015) demonstrated transfer of neurofeedback training and showed that activity within the IFG during motor imagery and motor execution of swallowing decreased (HbR increased) after neurofeedback training.

Some studies analyzed brain connectivity during neurofeedback training. Hudak et al. (2018) identified differential brain connectivity patterns for successful and failed regulation of the dlPFC, and demonstrated the importance of the fronto-parietal control network. Results may be specific to the training protocol and online analysis (i.e., influence of reference channels punishing activation in certain brain regions). However, Trambaiolli et al. (2018) found similar connectivity patterns for the random-, real-, and fixed-feedback (i.e., no-feedback) condition during an affective neurofeedback task.

### 4.3. Conclusion—Neural Effects of Neurofeedback

All in all, the results of some high-quality studies demonstrate the effectiveness of fNIRS-neurofeedback for regulating brain activation in motor regions (three of the successful studies) and in the OFC (one study). For other brain regions, such as the dlPFC, results are mixed and we cannot conclude whether fNIRS-neurofeedback is effective for regulating these regions as well. Moreover, a lacuna in reporting regulation success is evident as well, which, together with a high level of degrees of freedom on the part of the researchers as outlined above, raises suspicions of selectively reporting positive results (Simmons et al., 2011) and inflated effect sizes (Ioannidis, 2008). Furthermore, initial analyses provide preliminary evidence for potential neuroplastic effects of fNIRS-neurofeedback and further mechanistic insights. Future studies should follow up on such efforts and investigate neural mechanisms of neurofeedback. Neuroplastic effects may also be investigated in combination with other methods such as fMRI, which has higher spatial resolution and covers subcortical brain regions that contribute to neurofeedback learning (Emmert et al., 2016; Sitaram et al., 2017)

## 5. Behavioral Effects of fNIRS-Neurofeedback in Healthy and Clinical Populations

The ultimate goal of many neurofeedback applications is to induce significant effects in behavior as a prerequisite for developing clinical applications or neuroenhancing procedures. Because most studies have targeted prefrontal (*N* = 13) or motor brain regions (*N* = 7; see Figure 3B), we here review the effectiveness for improving executive functioning and motor rehabilitation across patient and healthy populations (see Table 7).

**Table 7:** Behavioral effects of fNIRS-neurofeedback.

**Study**	**Target region**	**Target population**	**Behavioral, cognitive/emotional effects**
Aranyi et al. (2016)	dlPFC asymmetry	18 healthy	Alignment ratings (the subjective rating of how appropriate the virtual agent's facial expressions were to the subject's thoughts during NF) correlated with regulation success (within)
Fujimoto et al. (2017)	SMA	20 healthy	Significant time × group interaction for postural control, but no time effects for both groups—interaction was mainly driven by decreased performance in the sham feedback group
Hosseini et al. (2016)	dlPFC	20 healthy	Improved working memory (significant time × group interaction). Exploratory: improved task switching
Hudak et al. (2017)	Bilateral dlPFC/IFG	20 highly impulsive	Reduction in false alarms (Go-NoGo task, within effect, only trend for interaction). Reduction in stop-signal reaction times (SSRT) variability (between effect), but no effect on SSRTs
Kimmig et al. (2018)	Bilateral dlPFC/IFG	12 SAD	Decreased social and general trait anxiety as well as depressive symptoms and a reduced disturbance of daily life. Improved social threat-processing, i.e., reduction of social threat-related attention bias toward laughter, but not specifically for taunting vs. joyful laughter. No correlation of behavioral effects with regulation performance
Li et al. (2019)	Right lateral OFC	60 healthy	Trend for enhanced cognitive flexibility (but only group comparison at post). Shorter response times and higher rewarding experience were associated with stronger training-induced HbO increases in lOFC
Liu et al. (2016)	Frontal and temporal face processing regions	2 healthy, 2 ASD	Improved facial recognition in all participants (single case analysis, no statistic reported)
Marx et al. (2015)	Bilateral dlPFC/IFG	27 children ADHD/9 per group	Decreased ADHD scores (parent and teacher ratings) within group. No effect on associated behavioral symptoms (SDQ) and quality of life (child ratings). Go-NoGo RTs, RT variability and commission errors decreased within group. But RT effect only from post to follow up. TAP flexibility RTs and RT variability decreased within group. In the control groups, ADHD scores did not significantly decrease, but also no significant group effect was found. Baseline differences in quality of life and associated behavioral symptoms
Mihara et al. (2012)	Left premotor cortex	21 healthy	Increased self-assessed kinesthetic motor imagery scores
Mihara et al. (2013)	Ipsilesional premotor cortex	20 stroke patients	Improved recovery of sensorimotor function, as assessed by Fugl-Meyer assessment scale (significant time × group Interaction), no significant adverse effect
Narita (2015)	Left PFC	4 ASD	Improved working memory, performance in Stroop task, anxiety and mood (within-effect, only means, no statistic reported)
Weyand et al. (2015)	Bilateral PFC	10 healthy	Marginal decrease in task load comparing session 10 (strategy use) and 15 (voluntary self-regulation). Work load decreased and ease-of-use and perceived intuitiveness only increased over self-regulation sessions (11–15), not over mental task sessions (1–10)

*ADHD, attention-deficit/hyperactivity disorder; ASD, autism spectrum disorder; dlPFC, dorsolateral prefrontal cortex; IFG, inferior frontal gyrus; OFC, orbitofrontal cortex; PFC, prefrontal cortex; SAD, social anxiety disorder; SMA, supplementary motor area*.

### 5.1. fNIRS-Neurofeedback to Improve Executive Functioning

Hosseini et al. (2016) found mixed effects on working memory after task-based neurofeedback training targeting down-regulation of the dlPFC. Performance improved in an n-back task as compared to the sham-feedback group, but no improvement was found in either group for the delayed verbal working-memory task used for the task-based neurofeedback paradigm. Additionally, a positive effect on task switching (analyzed exploratory) was reported. It should be noted that this study only reported marginal effects for regulation performance. Unfortunately, Barth et al. (2016) did not report the behavioral effects for working memory. In a subclinical sample, Hudak et al. (2017) found no effect in an n-back task but improved inhibitory control as assessed with a Go-NoGo task, and only a trend compared to an EMG-biofeedback control group. Stop-signal reaction-time variability also decreased (significant group effect). The latter study also does not show any indications of successful regulation. Li et al. (2019) found a trend for enhanced cognitive flexibility as assessed with an attentional set-shifting task, but they did not apply a pre-measurement and compared groups only at post. Moreover, OFC regulation correlated with reward experience in the neurofeedback group only.

Due to its potential to improve prefrontal brain functions, particularly inhibitory control, fNIRS-neurofeedback has been investigated as a potential treatment for children (Marx et al., 2015) and adults (Hudak et al., 2018) with ADHD. Indeed, Marx et al. (2015) found indications of improved inhibitory control after neurofeedback in children with ADHD as assessed with a Go-NoGo task, which was unfortunately not assessed in the control groups. Furthermore, this was accompanied by a decrease in ADHD symptoms, but similar improvements were also observed in the two active control groups. Therefore, while fNIRS-neurofeedback may improve attention and inhibitory control, leading to decreased ADHD symptoms, specificity has not been demonstrated yet and it remains open whether it offers any advantages over classical EEG-neurofeedback or other established treatments for ADHD. Larger clinical fNIRS-neurofeedback trials in children and adults with ADHD are currently under way (Mayer et al., 2015; Blume et al., 2017) and may shed further light on this issue.

Another potential clinical application for fNIRS-neurofeedback of the dlPFC is the treatment of social anxiety disorder. For example, it has been reported to reduce social threat-related attention bias and improve social and general trait anxiety as well as depressive symptoms (Kimmig et al., 2018). However, due to the absence of a control group, results are only preliminary. In addition, few indications of successful regulation were reported.

### 5.2. fNIRS-Neurofeedback for Motor Rehabilitation

Two studies demonstrated the modulation of swallowing-related motor regions (within the IFG) in healthy participants. HbR increased during motor imagery and execution of swallowing after training, though not compared to a control group. This training protocol might be investigated in patients with dysphagia in the future (Kober et al., 2015, 2018). fNIRS-neurofeedback of premotor regions improved self-assessed kinesthetic motor imagery during real neurofeedback as compared to a within sham-feedback condition in healthy participants (Mihara et al., 2012), but did not improve postural stability after one session (Fujimoto et al., 2017). The significant interaction was mainly driven by a decrease in postural stability after sham feedback. This missing effect might be attributed to the limited duration of training or to ceiling effects in the healthy participants, as a longer training period in patients after stroke was efficacious (Mihara et al., 2013). In this double-blind randomized sham-controlled design, patients underwent stroke rehabilitation and were trained to upregulate activation of the ipsilesional premotor cortex via motor imagery. The control group also practiced motor imagery but received artificially generated feedback. After six sessions specific effects were observed for the hand/finger subscale of the Fugl-Meyer assessment. Results are promising and indicate that fNIRS-neurofeedback may facilitate motor recovery in patients after stroke, but replications in a larger, controlled clinical trial are needed.

### 5.3. Other Potential Clinical Applications

FNIRS-neurofeedback was investigated as a potential treatment for autism spectrum disorder. Liu et al. (2016) applied a task-based neurofeedback approach to enhance the effects of facial-recognition training in adolescents with autism and found improved facial recognition which was also present in the patient receiving sham feedback. Given that these are only the initial data of a larger clinical trial which do not permit a statistical analysis, conclusions can be made only when all the data are published. Similarly, Narita (2015) trained four participants with autism to upregulate activity of the prefrontal cortex and reported improvements in working memory, inhibitory control as well as in anxiety and mood on a single-case level.

Furthermore, studies demonstrated successful classification of neutral and positive affective states (Trambaiolli et al., 2018) and regulation of asymmetric activation of the left dlPFC, a candidate neural mechanism of approach-avoidance motivation (Aranyi et al., 2016). These studies in healthy participants may pave the way for future applications in mood disorders.

Although not included in our review, it is worth mentioning a single-case study (Storchak et al., 2018) describing a new neurofeedback protocol to treat auditory verbal hallucinations in schizophrenia. A patient with paranoid schizophrenia was trained for 47 sessions to regulate the activity of the bilateral posterior superior temporal gyrus. To counteract neural correlates of auditory hallucinations, the patient was instructed to upregulate when expecting and downregulate when experiencing hallucinations. She was successful in upregulation, but not downregulation. However, even though amplitudes did not differ significantly from zero over the sessions, a learning effect was reported for downregulation, i.e., a significant decrease in activation over the sessions. Throughout the training period hallucinations decreased and symptoms improved.

### 5.4. Conclusion—Behavioral Effects of fNIRS-Neurofeedback in Healthy and Clinical Populations

In sum, there is preliminary evidence for the effectiveness of fNIRS-neurofeedback for improving motor rehabilitation and executive functions, particularly for improved inhibitory control (Marx et al., 2015; Hudak et al., 2017) and cognitive flexibility (Hosseini et al., 2016; Li et al., 2019). Mixed results were reported for working-memory tasks, which may be attributable to ceiling effects (Hudak et al., 2017). This is in line with the EEG-neurofeedback literature where similar effects were observed for inhibitory control tasks (Bluschke et al., 2016; Mayer et al., 2016). However, non-specific factors (psychosocial/placebo effects) may explain a large proportion of the effect sizes found in neurofeedback studies (Thibault and Raz, 2016; Schönenberg et al., 2017; Ros et al., 2020). Hence, due to the limited evidence available and a lack of well-powered (see section 3.3) properly sham-controlled studies, it remains difficult to make any claims about the specificity of the reported effects. Regarding clinical potential, early pilot studies show the feasibility of fNIRS-neurofeedback in different patient populations such as ADHD, social anxiety disorder, autism, and stroke. The most promising data are found for stroke rehabilitation, where a double-blind sham-controlled study demonstrated beneficial effects (Mihara et al., 2013). It should be noted that most studies investigated effects in healthy populations and stronger effects may be expected in patient populations, displaying more room for improvement. Great optimism has been expressed with regard to future clinical applications (Ehlis et al., 2018), but larger well-controlled studies and clinical trials are needed to corroborate initial findings and demonstrate specificity before fNIRS-neurofeedback can be considered a viable complementary or even alternative treatment option.

## The Potential of fNIRS for Neurofeedback Research—Future Directions

Neurofeedback research using fNIRS has just begun. Being more precise in targeting localized brain regions than EEG and much easier to use, and less expensive than fMRI, fNIRS may become an important tool for neurofeedback research and application. In this section, we outline our perspective on the future of fNIRS-neurofeedback and highlight its future potential.

Future research could benefit from exploiting the advantages of fNIRS to an even greater extent, since it offers unique opportunities for neurofeedback research. Compared to fMRI, it is easier to conduct a greater number of sessions and/or recruit more participants. This will help to solve the issue of low statistical power, which is a common problem in neuroscientific research (see section 3.3.4). Furthermore, this makes this technique more suitable than fMRI for larger multicenter studies. To our knowledge, no clinical multicenter fMRI-neurofeedback study has been published, while in EEG-neurofeedback research it has already been demonstrated that multicenter studies are possible (e.g., Strehl et al., 2017). Methods applied in studies are still quite heterogeneous and further agreements and standardization of protocols are necessary before this step can be taken. If the target is a region of the neocortex, it is also conceivable that methods may be combined, and successful fMRI-neurofeedback protocols transferred to fNIRS-neurofeedback to conduct a high-powered study employing many sessions and/or participants. Future studies could also compare fNIRS-based with fMRI-based neurofeedback protocols using simultaneous measures to reveal commonalities and differences or even combine both methods to exploit optimally their advantages. For example, in a first fMRI session the regions of interest could be precisely defined on the individual level to guide the placement of optodes (channels of interest) and improve the spatial specificity for the following (more economic) fNIRS-neurofeedback sessions.

FNIRS is particularly suited for and has been extensively and successfully used in developmental neuroscience (Lloyd-Fox et al., 2010; Pinti et al., 2018b). Future research may benefit from using fNIRS-neurofeedback particularly in children and older healthy and patient populations. These populations show more movement during experiments (e.g., Poldrack et al., 2002) and such movements are more tolerable with fNIRS. They also possess physical features that may be beneficial for fNIRS signal quality (e.g., skull thickness, hair thickness/pigmentation; Orihuela-Espina et al., 2010) and hence for neurofeedback applications.

Recently, portable and wireless fNIRS devices have been developed (Pinti et al., 2018b) that can be used outside the laboratory. Such portable devices could be used to conduct neurofeedback-training studies at home, in school, or any other place in the world, including also low-resource countries (Pinti et al., 2018b). If the ease-of-use of these devices were to be further developed participants could even conduct neurofeedback training on their own and thus train whenever they want. This would make it easy to conduct neurofeedback studies with long training times at low costs. Of course, before moving out of the laboratory, standard protocols have to be developed and proven to be effective.

Also, fNIRS may be particularly suitable for interactive hyperscanning neurofeedback approaches, which have already been introduced by Duan et al. (2013), who had two participants competing in a “tug-of-war” neurofeedback game. This competitive context may increase motivation. In the future, cooperative approaches may be introduced as well, where two or more participants regulate a target brain signal together instead of competing.

Recent developments in fMRI-neurofeedback protocols could be transferred to fNIRS-neurofeedback. To date none of the fNIRS-neurofeedback studies used connectivity measures as a source of neurofeedback (other than support vector machine-based techniques). Connectivity-based neurofeedback has already been developed for fMRI-neurofeedback (Koush et al., 2015; Spetter et al., 2017; Yamashita et al., 2017; Zhao et al., 2019). Generally, some of these methods could be transferred to fNIRS since the sources and detectors can be freely positioned and might allow similar regions to those used in fMRI-neurofeedback studies to be covered. The high sampling of fNIRS allows stable correlations to be estimated in potentially shorter time windows, which is an advantage in this regard. However, these protocols should be established with caution and only with proper control of extracranial artifacts (e.g., using partial correlation and/or short-distance channels), which may easily produce spurious correlations. Future studies could also be oriented to recent methodological developments in fMRI-neurofeedback, such as implicit/covert neurofeedback protocols [i.e., neurofeedback without participants' awareness (e.g., Ramot et al., 2017) or decoded neurofeedback (Shibata et al., 2019)] and process-based neurofeedback, where protocols are designed to target disorder-specific processes (Lubianiker et al., 2019). Also, fMRI-informed approaches could be explored, as already applied in EEG-neurofeedback (e.g., Meir-Hasson et al., 2016), where fNIRS channels would predict the fMRI signal of a target region. Such approaches, if feasible with fNIRS, could result in improved spatial specificity and ideally make it possible to assess subcortical brain regions.

The problem of not reporting important information about signal-processing methods is also clearly visible in fMRI-neurofeedback studies and was addressed in a recent review by Heunis et al. (2020) recommending more rigorous reporting and development of methodological reporting standards. These recommendations can also be applied to fNIRS-neurofeedback research and similar default measures such as signal- or contrast-to-noise ratio calculations for evaluating fNIRS-signal quality could be established to make results more comparable across studies and to improve reproducibility (Heunis et al., 2020). Notably, fMRI-neurofeedback can already rely on a further developed field, and efforts with regard to standardization have already been made (Nichols et al., 2016). This is not the case for fNIRS research, where even standards for offline analysis methods are still lacking (Kamran et al., 2016; Pinti et al., 2018b). However, discussing this issue now will help to improve reporting quality and reproducibility at an early stage.

In general, the field will benefit from adopting more rigorous research and reporting practices as encouraged by a recent consensus (Aczel et al., 2019; Ros et al., 2020) to improve the likelihood of replicability and reproducibility (Mehler, 2019). These measures include sampling plans that are ideally based on adequate power analyses which render both positive and negative findings more informative (Mehler et al., 2019). We acknowledge that it remains challenging to define the smallest effect sizes of interest, which are context-specific. We thus recommend that researchers explore different approaches which have been established to define SESOIs (Lakens et al., 2018). We also acknowledge that neurofeedback studies are very resource-intensive requiring several training sessions and hence sampling plans that require relatively large sample sizes may be unrealistic to implement, particularly when working with patients. We therefore recommend considering ways of collaboration including multilab studies or multicenter trials and exploring alternatives such as sequential sampling methods with flexible stopping that can yield higher sensitivity (Schönbrodt and Wagenmakers, 2018) and encourage transparency in reporting sampling plans (e.g., mentioning practical constraints). Researchers can efficiently document design decisions, including the sampling and planned analyses, by publishing their protocols. These additional publishing formats include preregistration, where researchers document their methodology with a timestamp on a public platform such as the Open Science Framework before data acquisition starts (e.g., Mehler et al., 2017). An alternative approach is to publish trial protocols in dedicated journals, which may be undertaken in parallel to data acquisition (e.g., Cox et al., 2016). Moreover, the recently introduced publishing format Registered Reports includes an initial peer-review stage that can grant authors acceptance in principle for their work independent of the statistical outcome. We note that adopting such methods is challenging: they involve additional costs such as more time in the preparation phase of the study and less flexibility (Allen and Mehler, 2019). However, recent preliminary meta-research suggests that the chances of publishing findings that do not meet traditional statistical thresholds increases remarkably for studies published as Registered Reports (Allen and Mehler, 2019; Scheel et al., 2020) while citation counts are comparable to traditional papers (Hummer et al., 2019). Hence, increased transparency not only benefits the field, but likely also individual authors. We therefore recommend that researchers should consider these publishing formats for future studies.

Lastly, it will be crucial to further develop standards and agreements, particularly for neurofeedback success measures, in order to have comparable outcome variables in the future (Haugg et al., 2020). We repeat suggestions by Paret et al. (2019) that a basic science approach should be employed, systematically exploring and optimizing neurofeedback protocols and real-time signal-processing methods, which can then inform translational work in the field.

## Conclusion

The present systematic review of fNIRS-neurofeedback studies suggests, although tentatively, that people can regulate hemodynamic signals from different cortical brain regions with fNIRS-neurofeedback indicating the feasibility of modulating normal behavior and psychiatric and neurological conditions. However, the field is at an early stage and consists mostly of feasibility, pilot, or proof-of-concept studies, so that the current systematic review might help to optimize future neurofeedback study designs but cannot provide recommendations on what neurofeedback targets, populations, and training protocols have proven most beneficial. There is room for improvement in reporting important information and statistical power, which impedes valid conclusions about specific behavioral effects or potential clinical utility of the method.

Nevertheless, fNIRS is becoming a viable method for neurofeedback research and has the potential for clinical translation of neurofeedback. Along this avenue, further methodological improvements, particularly aiming at improving signal quality, are of crucial importance and, together with more rigorous research and reporting practices, may improve the chances of replicability and reproducibility. This will help to gain a more solid understanding of fNIRS-neurofeedback and move the field closer toward agreements and standardization. FNIRS-neurofeedback is still in its infancy, and we now have the chance to create a solid foundation to build upon in the future. With this systematic review, we hope to stimulate a discussion about methodological and reporting standards at this early stage.

## Data Availability Statement

Data from this systematic review are available in the Supplementary Material as well as at the Open Science Framework (see https://osf.io/hnxfq/), where we plan to update the database regularly in the future.

## Author Contributions

SK conceptualized the review with input from all other authors, selected the studies, extracted the data, conducted the analysis, assessed the quality of the studies, drafted, and revised the manuscript. DM assessed the quality of the studies and revised the manuscript. ML, RT, and KK revised the manuscript. BS supervised the drafting of the manuscript and revised it. All authors contributed to the article and approved the submitted version.

## Conflict of Interest

ML is an employee of the research company Brain Innovation B.V., Maastricht, Netherlands. He was not involved in data extraction and quality assessments of the studies. He mainly contributed to the methodological section summarizing the current state of methods used for fNIRS real-time processing and discussing recent methodological developments in the field. RT has received payments to consult with neurofeedback start-up companies. The remaining authors declare that the research was conducted in the absence of any commercial or financial relationships that could be construed as a potential conflict of interest.

## Footnotes

^1^To simplify the text, in the following we subsume the conceptually different behavioral, cognitive, and emotional outcome variables under the general term *behavior*.
