# Corrigendum: The Potential of Functional Near-Infrared Spectroscopy-Based Neurofeedback—A Systematic Review and Recommendations for Best Practice

**DOI:** 10.3389/fnins.2022.907941

**Published:** 2022-08-22

**Authors:** Simon H. Kohl, David M. A. Mehler, Michael Lührs, Robert T. Thibault, Kerstin Konrad, Bettina Sorger

**Affiliations:** ^1^JARA-Institute Molecular Neuroscience and Neuroimaging (INM-11), Jülich Research Centre, Jülich, Germany; ^2^Child Neuropsychology Section, Department of Child and Adolescent Psychiatry, Psychosomatics and Psychotherapy, Medical Faculty, RWTH Aachen University, Aachen, Germany; ^3^Department of Psychiatry, University of Münster, Münster, Germany; ^4^Brain Innovation B.V., Research Department, Maastricht, Netherlands; ^5^Faculty of Psychology and Neuroscience, Department of Cognitive Neuroscience, Maastricht University, Maastricht, Netherlands; ^6^School of Psychological Science, University of Bristol, Bristol, United Kingdom; ^7^MRC Integrative Epidemiology Unit, University of Bristol, Bristol, United Kingdom

**Keywords:** real-time data analysis, functional near-infrared spectroscopy, neurofeedback, systematic review, clinical translation, self-regulation, brain-computer interfacing

In the original review article, we miscalculated the statistical power and sensitivity of the included studies which led to an overestimation of power and sensitivity. The miscalculations arose from an easy-to-miss default option for repeated measures (mixed) ANOVAs in the statistical software program GPower (Faul et al., 2007), which co-author (RTT) became aware of after our review was published.

The default option defines a variable in the effect size calculation (η2p) in such a way that the common usage of small, medium, and large effects sizes for the interaction of repeated measures (mixed) ANOVAs (f) do not hold true. If using the default option, the power calculations will account for the correlations between repeated measures a second time, and in turn erroneously increase power. In fact, the G^*^Power software itself recommends another option (“as in Cohen, 1988 – recommended”). We recalculated the statistical power and sensitivity of the included studies using the recommended effect-size estimation according to Cohen (1988), instead of the default option^1^.

Furthermore, we noticed a few other errors in the sensitivity and power analyses which we corrected:

(1) For the calculations, we previously used the sample size for all participants included in the study. We now use the sample size that the studies used in their statistical analysis.
(2) We removed the study by Weyand et al. (2015) from the analysis because it only ran binomial tests within each participant but did not test for group effects.
(3) A typo in Table S3 depicting the statistical power for the study of Mihara et al. (2012).
(4) We now use independent *t*-tests instead of paired t-tests for two studies in the analysis as depicted in Table S5.

Below we rewrite specific paragraphs from our review where our power and sensitivity recalculations have changed the values presented.

A correction has been made to Section 3, Quality of published studies, 3.3 Statistical Power/Sensitivity, Paragraph 5 and 6. As a result of the recalculations, we updated the values in this section, which now reads as follows:

“Overall, sample sizes varied across studies from our minimum inclusion size, i.e., four (two per cell), up to 60 (30 per cell). One single group study included 33 participants. The median sample size of the studies was 20 (12 per cell). We calculated the median effect size that was detectable with 80 and 95% power. For the outcome regulation performance, we found a value of *d* = 0.85 and *d* = 1.13, respectively. For behavioral outcomes, the value was *d* = 1.28 and *d* = 1.63, respectively. We further found that the median power to detect a small effect of *d* = 0.2 was low (0.14 and 0.08). Our results showed that the power was even insufficient to reliably detect large behavioral effects (0.43). Studies were only powered to reliably detect large effects in regulation performance (0.75; see Table 5 and **Tables S3**, **S4** for more details).

Some studies that included control groups lacked a direct statistical comparison of regulation performance between the experimental and the control condition. Instead, these studies only reported the main effects within conditions and compared statistical significance between conditions instead of effect sizes. This statistical approach is erroneous for group comparisons (Nieuwenhuis et al., 2011) and makes it difficult to assess whether the experimental group outperformed the control group. If we assume that these studies found no statistically significant group effect, we note that due to insufficient statistical power we cannot come to valid conclusions about potential group effects. To check this assumption, we conducted sensitivity analyses for the respective group/interaction effects, using a statistical test that was appropriate for the respective study design (see **Supplementary Material**). Sensitivity for detecting a certain group/interaction effect size was lower as compared to detecting a within-group main effect. It is reasonable to assume that interaction effects are smaller and, depending on the underlying assumptions, require four to sixteen times more participants to achieve similar a priori statistical power (Gelman, 2018). Therefore, studies were likely underpowered for reliably detecting group differences in neurofeedback effects, which are very likely smaller than within-group effects.”

A correction has been made to Supplementary Material, Section 5, Comparison of Sensitivity of Regulation Performance Analysis of Studies not Comparing the Effect to the Control Condition/Group, Paragraph 1 and 2. As a result of the recalculations, we updated the values in this section, which now reads as follows:

“Here we report detailed results of the comparison of sensitivity for tests reported by studies comparing regulation performance to baseline and sensitivity for a hypothetical comparison with a control group/condition. We used G^*^Power (Faul et al., 2007) to calculate and compare the sensitivity of the baseline comparison as reported by the study and the hypothetical group comparison using a statistical test that was appropriate for the respective study design. **Table S5** shows detailed results.

Results showed that the sensitivity for estimating a group/interaction effect as compared to a within-group effect was lower. In placebo-controlled treatment studies (e.g., sham neurofeedback training) effect sizes in the control group constitute a large portion of the effect size of the active group (Wampold et al., 2005) and large effects have been reported for sham neurofeedback, at least in the context of EEG-nf (Schönenberg et al., 2017). Therefore, we can assume that an interaction effect testing for group differences over time is smaller than the main effect within groups, except for studies that successfully employ a bidirectional control where participants of both groups learn to regulate a brain signal in opposite directions. Hence, for most cases we can assume that interaction effects are a fraction of main effects and require substantially larger sample sizes to achieve similar statistical power. To further illustrate this, we can consider a classical neurofeedback study design consisting of a 2 × 2 within-between subject design (ANOVA approach) and assume an effect size of *d* = 0.4 for the main effect and half that size *d* = 0.2 for the interaction effect. According to an analysis with G^*^Power, such a design needs a total sample size of at least 200 participants to detect the main effect at 80% power, and almost four times as many participants (*N* = 788) to detect the comparatively small interaction effect. We further note that under different statistical assumptions the required sample size to detect the interaction effect is as much as sixteen times greater (see Gelman, 2018). Hence, studies were underpowered to reliably detect group differences, which are very likely smaller than within-effects.”

A correction has been made to the Supplementary Tables as follow:

**Table S3**. Sensitivity and statistical power for reported analysis of regulation performance.

The table shows the updated values of the recalculation of the sensitivity and statistical power. The study of Weyand et al. (2015) which was excluded from the analysis has been removed. No other changes have been made to the table.

**Table S4**. Sensitivity/power analysis for behavioral effects.

The table shows the updated values of the recalculation of the sensitivity and statistical power. No other changes have been made to the table.

**Table S5**. Comparison of sensitivity of regulation performance analysis of studies not comparing the effect to the control condition/group.

The table shows the updated values of the recalculation of the sensitivity and statistical power. No other changes have been made to the table.

In the original article, as a result of the erroneous analyses, there were mistakes in:

Figure 4. Statistical power curves to detect different effect sizes with 20 participants (median sample size) for different statistical tests.

Compared to the original review article only the power curve for the 2x2 Mixed ANOVA has changed which now displays the decreased statistical power according to the recalculation.

Table 5. Sensitivity and statistical power for reported analysis.

The table shows the updated values of the recalculation of the sensitivity and statistical power. No other changes have been made to the table.

The corrected Figure and Table appear below.

**Figure 4:**
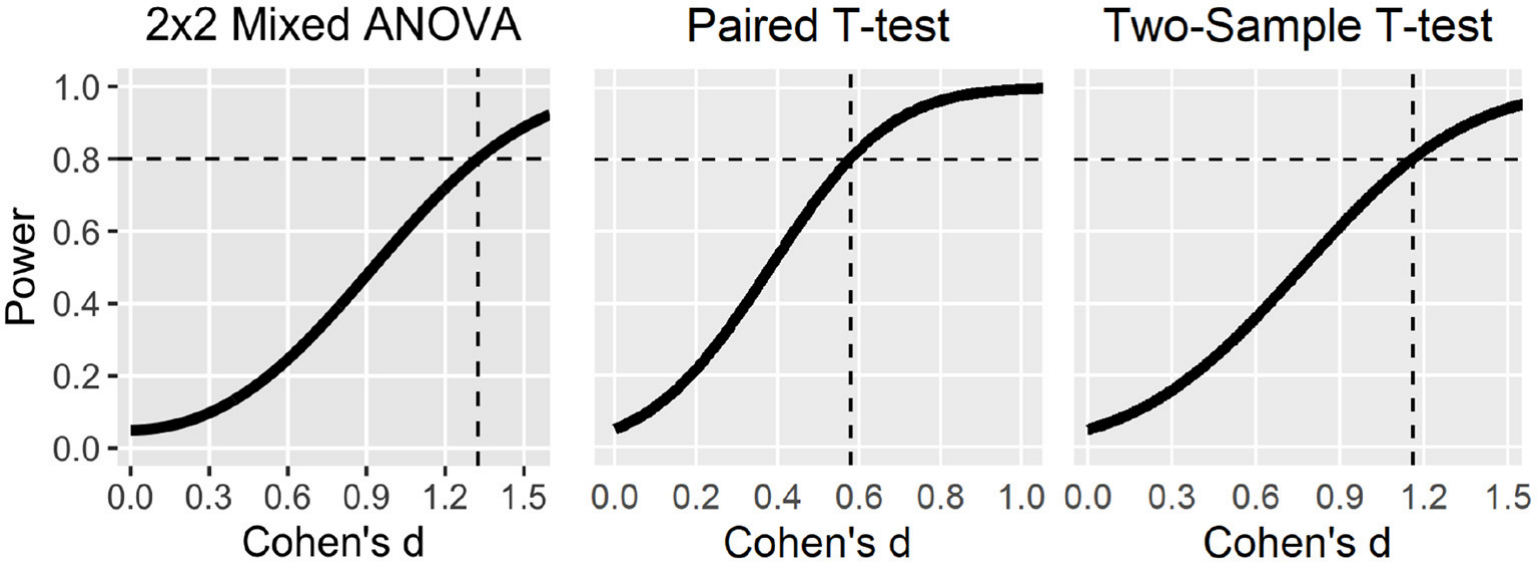
Statistical power curves to detect different effect sizes with 20 participants (median sample size) for different statistical tests. Dashed lines indicate smallest effect sizes detectable at 80% power. Note that the power curve for the 2 × 2 mixed ANOVA was based on liberal statistical assumptions (e.g., high correlation among repeated measures, sphericity, and uncorrected *p*-value of 0.05).

**Table 5:** Sensitivity and statistical power for reported analysis.

**Study**	***N***	**Sensitivity**		**Power to detect**		
		**80% power**	**95% power**	***d* = 0.2**	***d* = 0.5**	***d* = 0.8**
**Regulation performance**						
Mean	19.29	*d* = 1.06	*d* = 1.38	0.14	0.41	0.66
Median	19	*d* = 0.85	*d* = 1.13	0.14	0.43	0.75
**Behavioral outcomes**						
Mean	22.1	*d* = 1.11	*d* = 1.45	0.10	0.31	0.56
Median	20	*d* = 1.30	*d* = 1.66	0.08	0.22	0.42

*Note that in order to simplify the analysis for some studies, we performed the analysis for a different statistical test than originally reported, did not take into account correction for multiple comparisons, and assumed no sphericity violation and a high correlation among repeated measures for ANOVAs. Overall, these measures should have led to an overestimation of the statistical power/sensitivity of the studies*.

Importantly, the sensitivity and power analyses in the original review article were already based on liberal statistical assumptions (e.g., high correlation among repeated measures, sphericity, and uncorrected p-value of 0.05), which should have led to an overestimation of the statistical power/sensitivity of the studies as already stated in the original review article (see page 18–20). The miscalculations as described only magnify the already existing overestimation but do not substantially alter the conclusions of the original review article, in which we state a lack of statistical power and sensitivity to detect realistic effect sizes (see page 18–20 in the original article).

The original article has been updated.

## Publisher's Note

All claims expressed in this article are solely those of the authors and do not necessarily represent those of their affiliated organizations, or those of the publisher, the editors and the reviewers. Any product that may be evaluated in this article, or claim that may be made by its manufacturer, is not guaranteed or endorsed by the publisher.

## Footnotes

^1^The text in this paragraph was adapted from the commentary of Thibault and Pedder (2022).
