# Supplementary material for: The Potential of Functional Near-Infrared Spectroscopy-Based Neurofeedback—A Systematic Review and Recommendations for Best Practice
Source: Front Neurosci. 2020 Jul 21;14:594. doi: 10.3389/fnins.2020.00594 (PMC7396619; doi:10.3389/fnins.2020.00594)
Supplement: Supplementary file 1 [file Table_1.docx]

Supplementary Material

This document provides supplementary material pertaining to the manuscript “The potential of functional near-infrared spectroscopy-based neurofeedback – a systematic review and recommendations for best practice”.

# Data extraction

# See Supplementary_file_1_Data_extraction.xlsx

1. **Details of JBI ratings**

To assess the methodological quality of the included studies we used the checklist for quasi-experimental studies of the Joanna Briggs Institute (JBI) critical appraisal tools (Tufanaru et al., 2017). Table S1 shows detailed results of the ratings. Here we further specify the criteria we applied for the ratings.

***Item 2: Similar participants in compared groups***

We assumed similar participants in terms of baseline measures or demographics, even if a study did not report a check for similarity. We acknowledge that studies reporting baseline differences more transparently (e.g., Hudak et al., 2017; Marx et al., 2015) may have been rated worse on this item.

***Item 3: Similar treatment in compared groups***

Only a few studies explicitly checked this assumption (e.g., Marx et al., 2015). If a study did not report any other intervention/exposures we assumed that no additional treatment was provided and that participants in both groups had similar exposure to other variables and thus rated “yes” for this item.

***Item 4: Existence of control group/condition***

All control conditions were rated “yes”. Within-subject control conditions had to be randomized.

***Item 5: Multiple measurement points of the outcome***

In this respect, we deviated from the JBI manual, which required multiple measurement time points of the outcome both before and after the intervention, i.e., at least four measurement points. Due to the novelty of the field and given the relatively large proportion of pilot studies, requiring four measurement points seemed too rigorous and hence we considered two measurement points to be sufficient (i.e., a classical pre-post design). Some studies were mainly interested in regulation performance and did not have a separate pre- and post-intervention measurement to assess behavioral changes in a pre-post design. We rated “yes” for those studies if the design comprised at least two sessions on two separate days.

***Item 6: Completion of follow-up***

We assessed whether the primary endpoint was completed. Studies did not have to include separate follow-up measurements after training to fulfill this item. For within-subject designs, we assessed whether there were any participants that took part in only one condition. If a study was mainly interested in regulation performance and did not have at least two separate training sessions on two different days, we rated this item as “not applicable”.

***Item 9: Appropriate statistical methods***

We rated this item “No”, if no power analysis was conducted and the study was not labeled *pilot, proof-of concept/proof-of-principle, or feasibility study*.

Table S1. Quality assessments of the included studies according to the checklist for quasi-experimental studies of the Joanna Briggs Institute (JBI) critical appraisal tools.

| **Study** | **1. Cause and effect** | | **2. Similar participants** | **3. Similar treatment** | **4. Control condition** | **5. Multiple measures** | **6. Follow up complete** | **7. Same outcome measures** | **8. Outcome reliability** | **9. Statistical methods** | **Total score** |
| --- | --- | --- | --- | --- | --- | --- | --- | --- | --- | --- | --- |
| Aranyi et al. (2016) | | Y | N/A | N/A | N | N | N/A | N/A | Y | N | 2 |
| Barth et al. (2016) | | Y | N/A | N/A | N | Y | N/A | N/A | Y | N | 3 |
| Fujimoto et al. (2017) | | Y | Y | Y | Y | Y | Y | Y | Y | N | 8 |
| Hosseini et al. (2016) | | Y | Y | Y | Y | Y | Y | Y | Y | N | 7 |
| Hudak et al. (2017) | | Y | N | Y | Y | Y | Y | Y | Y | Y | 8 |
| Hudak et al. (2018) | | Y | N/A | N/A | N | Y | N/A | N/A | Y | N | 3 |
| Kanoh et al. (2011) | | Y | N/A | N/A | N | Y | Y | N/A | Y | N | 4 |
| Kimmig et al. (2018) | | Y | N/A | N/A | N | Y | Y | N/A | Y | Y | 5 |
| Kinoshita et al. (2016) | | Y | Y | Y | Y | N | N/A | Y | Y | N | 6 |
| Kober et al. (2014) | | Y | Y | Y | Y | Y | Y | Y | Y | N | 7 |
| Kober et al. (2015) | | Y | Y | Y | Y | Y | Y | Y | Y | N | 8 |
| Kober et al. (2018) | | Y | N | Y | Y | N | N/A | Y | Y | N | 5 |
| Lapborisuth et al. (2017) | | Y | N/A | N/A | N* | N | N/A | N/A | Y | N | 2 |
| Lee et al. (2015) | | Y | N/A | N/A | U* | N | N/A | Y | Y | N | 3 |
| Li et al. (2019) | | Y | Y | Y | Y | N | N/A | Y | Y | Y | 7 |
| Liu et al. (2016) | | Y | U | U | Y | Y | Y | Y | Y | N/A | 6 |
| Marx et al. (2015) | | Y | N | Y | Y | Y | Y | Y | Y | Y | 8 |
| Mihara et al. (2012) | | Y | Y | Y | Y | N | N/A | Y | Y | N | 6 |
| Mihara et al. (2013) | | Y | Y | Y | Y | Y | Y | Y | Y | N | 8 |
| Narita et al. (2015) | | Y | N/A | N/A | N | Y | Y | N/A | Y | N | 4 |
| Trambaiolli et al. (2018) | | Y | Y | Y | Y | N | N/A | Y | Y | N | 6 |
| Weyand et al. (2015) | | Y | N/A | N/A | N | Y | Y | N/A | Y | N | 4 |
| Total | | 22 | 9 | 12 | 13 | 14 | 12 | 14 | 22 | 4 | M = 5.55  SD = 2.15 |

Y. Yes; N. No; U. Unclear. N/A. Not Applicable; note that in some cases we deviated from the JBI manual (see Supplementary Material for details). * Applied a within-control, but did not randomize order of conditions or did not report randomization.

# Details of CRED-nf ratings

To assess the experimental design and reporting quality of the included studies we used the CRED-nf checklist (Ros et al., 2020). Here we further specify the criteria we applied during the ratings. In some cases, we deviated from the original CRED-nf items and applied more lenient criteria. We note that this should be considered in future comparisons applying the CRED-nf checklist.

***Item 2b. When leveraging experimental designs where a double-blind is possible, use a double-blind***

We deviated from the original item and each study reporting any information about the blinding procedure was rated “yes”, including also single-blinded studies and studies discussing the absence of blinding.

***Item 4b. Report and justify the reinforcement schedule***

We did not consider justification of the reinforcement schedule necessary, but each study that at least reported feedback threshold criteria was rated “yes” on this item.

***Item 4d. Collect and report all brain activity variable(s) and/or contrasts used for feedback, as displayed to experimental participants***

Here we considered reporting of the essential time points (ii) rest blocks and (iii) training blocks to be sufficient if a study reported the contrast for regulation *vs.* rest for the channel(s) of interest used for feedback. Reporting or plotting main effects solely for the conditions of rest and regulation was not required.

***Item 5b. Plot within-session and between-session regulation blocks of feedback variable(s), as well as pre-to-post resting baselines or contrasts***

In this case we deviated from the original item, which requires plotting all three effects. Probably related to the novelty of the field, most studies did not apply pre-post resting baselines. In addition, there are several one-session designs that cannot plot any between-session effects. Hence, we rated “yes” if a study plotted time courses on at least one of the two temporal levels of within- or between-session.

Table S2. Reporting and design quality according to the CRED-nf checklist.

|  |  | | |  | **Study** | | | | | | | | | | | | | | | | | | | | | | |  | | |
| --- | --- | --- | --- | --- | --- | --- | --- | --- | --- | --- | --- | --- | --- | --- | --- | --- | --- | --- | --- | --- | --- | --- | --- | --- | --- | --- | --- | --- | --- | --- |
| **Domain** | | **Items** | | | | Aranyi et al. (2016) | Barth et al. (2016) | Fujimoto et al. (2017) | Hosseini et al. (2016) | Hudak et al. (2017) | Hudak et al. (2018) | Kanoh et al. (2011) | Kimmig et al. (2018) | Kinoshita et al. (2016) | Kober et al. (2014) | Kober et al. (2015) | Kober et al. (2018) | Lapborisuth et al. (2017) | Lee et al. (2015) | Li et al. (2019) | Liu et al. (2016) | Marx et al. (2015) | Mihara et al. (2012) | Mihara et al. (2013) | Narita et al. (2015) | Trambaiolli et al. (2018) | Weyand et al. (2015) | | **Total** |  |
| **Pre-experiment** | | 1a | Preregistration | | | 0 | 0 | 0 | 0 | 0 | 1 | 0 | 0 | 0 | 0 | 0 | 0 | 0 | 0 | 0 | 0 | 0 | 0 | 0 | 0 | 0 | 0 | | 1 |  |
|  | | 1b | Sampling plan | | | 0 | 1 | 0 | 1 | 1 | 0 | 0 | 1 | 0 | 0 | 0 | 0 | 0 | 0 | 1 | 1 | 1 | 0 | 1 | 0 | 0 | 0 | | 8 |  |
| **Control groups** | | 2a | Control condition | | | 0 | 0 | 1 | 1 | 1 | 0 | 0 | 0 | 1 | 1 | 1 | 1 | 1 | 1 | 1 | 1 | 1 | 1 | 1 | 0 | 1 | 0 | | 15 |  |
|  | | 2b* | Blinding | | | 0 | 0 | 1 | 0 | 1 | 0 | 0 | 0 | 1 | 1 | 1 | 1 | 0 | 0 | 1 | 0 | 0 | 1 | 1 | 0 | 0 | 0 | | 9 |  |
|  | | 2c | Blinding - rater | | | 0 | 0 | 0 | 0 | 0 | 0 | 0 | 0 | 0 | 0 | 0 | 0 | 0 | 0 | 0 | 0 | 0 | 0 | 1 | 0 | 0 | 0 | | 1 |  |
|  | | 2d | Blinding assessed | | | 0 | 0 | 0 | 0 | 0 | 0 | 0 | 0 | 0 | 0 | 0 | 0 | 0 | 0 | 0 | 0 | 0 | 1 | 0 | 0 | 0 | 0 | | 1 |  |
|  | | 2e | Standard of care intervention group | | | 0 | 0 | 0 | 0 | 0 | 0 | 0 | 0 | 0 | 0 | 0 | 0 | 0 | 0 | 0 | 0 | 1 | 0 | 1 | 0 | 0 | 0 | | 2 |  |
| **Control measures** | | 3a | Psychosocial factors assessed | | | 0 | 0 | 1 | 0 | 0 | 0 | 0 | 1 | 0 | 0 | 0 | 0 | 0 | 0 | 1 | 0 | 1 | 0 | 0 | 0 | 1 | 1 | | 6 |  |
|  | | 3b | Strategy provided | | | 1 | 0 | 1 | 1 | 1 | 0 | 1 | 0 | 1 | 1 | 1 | 1 | 1 | 0 | 1 | 1 | 0 | 1 | 1 | 0 | 1 | 1 | | 16 |  |
|  | | 3c | Strategies used | | | 1 | 1 | 0 | 0 | 0 | 0 | 0 | 0 | 0 | 0 | 1 | 0 | 0 | 0 | 1 | 0 | 0 | 0 | 0 | 0 | 1 | 1 | | 6 |  |
|  | | 3d | Online-data processing and artifact control | | | 1 | 0 | 1 | 1 | 1 | 1 | 1 | 1 | 1 | 1 | 1 | 0 | 1 | 0 | 1 | 1 | 1 | 1 | 1 | 0 | 1 | 1 | | 18 |  |
|  | | 3e | Effects of artifacts | | | 0 | 0 | 0 | 0 | 0 | 0 | 0 | 0 | 1 | 0 | 0 | 0 | 0 | 0 | 0 | 0 | 0 | 0 | 0 | 0 | 0 | 0 | | 1 |  |
| **Feedback specifications** | | 4a | Online feature extraction | | | 1 | 1 | 1 | 1 | 1 | 1 | 1 | 1 | 1 | 1 | 1 | 0 | 1 | 1 | 1 | 1 | 1 | 1 | 1 | 1 | 1 | 1 | | 21 |  |
|  |  | 4b* | Reinforcement schedule | | | 1 | 0 | 1 | 1 | 1 | 1 | 0 | 1 | 0 | 1 | 1 | 0 | 0 | 1 | 1 | 1 | 1 | 1 | 1 | 0 | 1 | 1 | | 16 |  |
|  |  | 4c | Feedback modality and content | | | 1 | 1 | 1 | 1 | 1 | 1 | 1 | 1 | 1 | 1 | 1 | 1 | 1 | 1 | 1 | 1 | 1 | 1 | 1 | 1 | 1 | 1 | | 22 |  |
|  |  | 4d* | Brain activity variables used for feedback | | | 1 | 1 | 1 | 1 | 0 | 1 | 1 | 0 | 1 | 1 | 1 | 1 | 1 | 1 | 1 | 1 | 0 | 1 | 1 | 0 | 1 | 1 | | 18 |  |
|  |  | 4e | Hardware and software | | | 1 | 1 | 1 | 1 | 1 | 1 | 1 | 1 | 1 | 1 | 1 | 1 | 1 | 1 | 1 | 1 | 1 | 1 | 1 | 1 | 1 | 1 | | 22 |  |
| **Outcome measures – Brain** | | 5a | Neurofeedback regulation success | | | 1 | 1 | 1 | 1 | 1 | 1 | 1 | 1 | 1 | 1 | 1 | 1 | 0 | 1 | 1 | 1 | 0 | 0 | 1 | 0 | 1 | 1 | | 18 |  |
|  |  | 5b* | Plots of feedback variable(s) | | | 0 | 1 | 1 | 1 | 0 | 0 | 0 | 0 | 0 | 1 | 1 | 1 | 0 | 0 | 1 | 1 | 0 | 0 | 1 | 0 | 0 | 1 | | 10 |  |
|  |  | 5c | Group comparison | | | 0 | 0 | 1 | 0 | 0 | 0 | 0 | 0 | 1 | 1 | 0 | 0 | 0 | 0 | 1 | 0 | 0 | 1 | 1 | 0 | 1 | 0 | | 7 |  |
| **Outcome measures - Behavior** | | 6a | Clinical or behavioral significance | | | 0 | 0 | 0 | 0 | 0 | 0 | 0 | 0 | 0 | 0 | 0 | 0 | 0 | 0 | 0 | 0 | 0 | 0 | 0 | 0 | 0 | 0 | | 0 |  |
|  |  | 6b | Correlation with behavioral outcomes | | | 0 | 0 | 0 | 1 | 1 | 0 | 0 | 1 | 0 | 0 | 1 | 0 | 0 | 0 | 1 | 0 | 0 | 1 | 1 | 0 | 1 | 0 | | 8 |  |
| **Data storage** | | 7a | Data storage | | | 0 | 0 | 0 | 0 | 0 | 0 | 0 | 0 | 0 | 0 | 0 | 0 | 0 | 0 | 0 | 0 | 0 | 0 | 0 | 0 | 0 | 0 | | 0 |  |
|  |  | | **CRED-nf essential** | | | 8 | 7 | 12 | 12 | 11 | 7 | 7 | 8 | 10 | 12 | 12 | 8 | 7 | 7 | 14 | 11 | 7 | 11 | 14 | 3 | 11 | 9 | |  |  |
|  |  | | **CRED-nf encouraged** | | | 1 | 1 | 1 | 0 | 0 | 1 | 0 | 1 | 1 | 0 | 1 | 0 | 0 | 0 | 2 | 0 | 2 | 1 | 2 | 0 | 2 | 2 | |  |  |
|  |  | | **CRED-nf total** | | | 9 | 8 | 13 | 12 | 11 | 8 | 7 | 9 | 11 | 12 | 13 | 8 | 7 | 7 | 16 | 11 | 9 | 12 | 16 | 3 | 13 | 11 | |  |  |

CRED-nf, Consensus on the reporting and experimental design of clinical and cognitive-behavioural neurofeedback studies checklist (Ros et al., 2019); 1 = reported; 0 = not reported. * We deviated from the original CRED-nf items and applied more lenient criteria (see text above).

# Sensitivity and statistical power

Here we provide detailed results of the analysis of statistical sensitivity and power of the included studies. Table S3 shows results for the analyses of regulation performance and Table S4 for the analyses of behavioral effects reported by the individual studies.

Table S3. Sensitivity and statistical power for reported analysis of regulation performance.

| **Study** | **N** | **Sample size for test** | **Sensitivity** | | | **Power to detect** | | |
| --- | --- | --- | --- | --- | --- | --- | --- | --- |
|  |  |  | **Test applied** | **80%** | **95%** | **d = 0.2** | **d = 0.5** | **d = 0.8** |
| Aranyi et al. (2016) | 18 | 17 | biserial correlation | d = 1.26 | d = 1.67 | 0.11 | 0.26 | 0.47 |
| Barth et al. (2016) | 13 | 13 | one-sample t-test (1-sided) | d = 0.73 | d = 0.97 | 0.17 | 0.52 | 0.86 |
| Fujimoto et al. (2017) | 20 | 20 | interaction of mixed-ANOVA (2x2) | d = 1.32 | d = 1.71 | 0.07 | 0.19 | 0.40 |
| Hosseini et al. (2016) | 20 | 10 | one-sample t-test on regression slopes (1-sided) | d = 0.85 | d = 1.13 | 0.15 | 0.43 | 0.75 |
| Hudak et al. (2017) | 20 | 10 | paired t-test (1-sided) | d = 0.85 | d = 1.13 | 0.15 | 0.43 | 0.75 |
| Hudak et al. (2018) | 19 | 19 | paired t-test (1-sided) | d = 0.59 | d = 0.79 | 0.21 | 0.67 | 0.96 |
| Kimmig et al. (2018) | 11 | 11 | paired t-test (1-sided) | d = 0.81 | d = 1.07 | 0.15 | 0.46 | 0.79 |
| Kinoshita et al. (2016) | 24 | 24 | rm-ANOVA (1 factor) | d = 1.39 | d = 1.74 | 0.06 | 0.15 | 0.33 |
| Kober et al. (2014) | 17 | 17 | ANCOVA | d = 1.46 | d = 1.88 | 0.07 | 0.16 | 0.34 |
| Kober et al. (2015) | 20 | 7 sessions * | linear regression | d = 2.66 | d = 3.46 | 0.06 | 0.09 | 0.14 |
| Kober et al. (2018) | 48 | 12 | one-sample t-test on regression slopes (1-sided) | d = 0.77 | d = 1.02 | 0.16 | 0.49 | 0.83 |
| Lapborisuth et al. (2017) | 22 | 22 | one-sample t-test (1-sided) | d = 0.55 | d = 0.73 | 0.23 | 0.73 | 0.98 |
| Lee et al. (2015) | 4 | 4 | one-sample t-test (1-sided) | d = 1.65 | d = 2.23 | 0.09 | 0.2 | 0.35 |
| Li et al. (2019) | 60 | 56 | mixed ANOVA 2x2 | d = 0.76 | d = 0.98 | 0.11 | 0.45 | 0.84 |
| Mihara et al. (2012) | 21 | 21 | one-sample t-test (2-sided) for contrast real vs. sham | d = 0.64 | d = 0.83 | 0.14 | 0.59 | 0.94 |
| Mihara et al. (2013) | 20 | 20 | unpaired t-test (1-sided?) | d = 1.16 | d = 1.53 | 0.11 | 0.28 | 0.53 |
| Trambaiolli et al. (2018) | 32 | 32 | paired t-test (1-sided) | d = 0.45 | d = 0.6 | 0.3 | 0.87 | 1 |
| **Mean** | 22.88 | 19.29 |  | d = 1.06 | d = 1.38 | 0.13 | 0.40 | 0.66 |
| **Median** | 20 | 19 |  | d = 0.85 | d = 1.13 | 0.14 | 0.43 | 0.75 |

* Kober et al. (2015) used the group averages for each session to calculate regression models (n = 7 sessions) for both groups separately (n = 10 subjects per group) and ignored subject variability. Therefore, we used n = 7 sessions instead of n = 10 participants.

Table S4. Sensitivity/power analysis for behavioral effects.

| **Study** | **N** | **Sample size for test** | **Test** | **Sensitivity** | | | **Power to detect** | | |
| --- | --- | --- | --- | --- | --- | --- | --- | --- | --- |
|  |  |  |  | **80%** | **95%** | **d = 0.2** | | **d = 0.5** | **d = 0.8** |
| Aranyi et al. (2016) | 18 | 17 | correlation (1-sided) | d = 1.37 | d = 1.91 | 0.10 | | 0.24 | 0.45 |
| Fujimoto et al. (2017) | 20 | 20 | interaction of mixed-ANOVA (2x2) | d = 1.32 | d = 1.71 | 0.07 | | 0.19 | 0.40 |
| Hosseini et al. (2016) | 20 | 20 | interaction of mixed-ANOVA (2x2) | d = 1.32 | d = 1.71 | 0.07 | | 0.19 | 0.40 |
| Hudak et al. (2017) | 20 | 20 | interaction of mixed-ANOVA (2x2) | d = 1.32 | d = 1.71 | 0.07 | | 0.19 | 0.40 |
| Kimmig et al. (2018) | 11 | 11 | paired t-test (1-sided) | d = 0.81 | d = 1.07 | 0.15 | | 0.46 | 0.80 |
| Li et al. (2019) | 60 | 56 | two-sample t-test (1-sided) | d = 0.67 | d = 0.89 | 0.18 | | 0.58 | 0.91 |
| Marx et al. (2015) | 27 | 27 | interaction of mixed-ANOVA (2x3) | d = 1.27 | d = 1.61 | 0.07 | | 0.18 | 0.40 |
| Mihara et al. (2012) | 21 | 21 | paired t-test (2-sided) | d = 0.64 | d = 0.83 | 0.14 | | 0.59 | 0.94 |
| Mihara et al. (2013) | 20 | 20 | interaction of mixed-ANOVA (2x2) | d = 1.32 | d = 1.71 | 0.07 | | 0.19 | 0.40 |
| Weyand et al. (2015) | 9 | 9 | paired t-test (2-sided) | d = 1.07 | d = 1.38 | 0.08 | | 0.26 | 0.56 |
| **Mean** | 22.6 | 22.1 |  | d = 1.11 | d = 1.45 | 0.1 | | 0.31 | 0.56 |
| **Median** | 20 | 20 |  | d = 1.30 | d = 1.66 | 0.08 | | 0.22 | 0.42 |

Note that in order to simplify the analysis, for some studies we performed the analysis for a different statistical test than the one originally reported, did not take into account correction for multiple comparisons, and assumed no sphericity violation and a high correlation among repeated measures for ANOVAs. Overall, these measures should have led to overestimation of statistical power/sensitivity of the studies.

1. **Comparison of sensitivity of regulation performance analysis of studies not comparing the effect to the control condition/group**

Here we report detailed results of the comparison of sensitivity for tests reported by studies comparing regulation performance to baseline and sensitivity for a hypothetical comparison with a control group/condition. We used G*Power (Faul et al., 2007) to calculate and compare the sensitivity of the baseline comparison as reported by the study and the hypothetical group comparison using a statistical test that was appropriate for the respective study design. Table S5 shows detailed results.

Results showed that the sensitivity for estimating a group/interaction effect as compared to a within-group effect was lower. In placebo-controlled treatment studies (e.g., sham neurofeedback training) effect sizes in the control group constitute a large portion of the effect size of the active group (Wampold et al., 2005) and large effects have been reported for sham neurofeedback, at least in the context of EEG-nf (Schönenberg et al., 2017). Therefore, we can assume that an interaction effect testing for group differences over time is smaller than the main effect within groups, except for studies that successfully employ a bidirectional control where participants of both groups learn to regulate a brain signal in opposite directions. Hence, for most cases we can assume that interaction effects are a fraction of main effects and require substantially larger sample sizes to achieve similar statistical power. To further illustrate this, we can consider a classical neurofeedback study design consisting of a 2×2 within-between subject design (ANOVA approach) and assume an effect size of d = 0.4 for the main effect and half that size d = 0.2 for the interaction effect. According to an analysis with G*Power, such a design needs a total sample size of at least 200 participants to detect the main effect at 80% power, and almost four times as many participants (N = 788) to detect the comparatively small interaction effect. We further note that under different statistical assumptions the required sample size to detect the interaction effect is as much as sixteen times greater (see Gelman, 2018). Hence, studies were underpowered to reliably detect group differences, which are very likely smaller than within-effects.

**Table S5.** Comparison of sensitivity of regulation performance analysis of studies not comparing the effect to the control condition/group.

| **Study** | **N** | **Sample size for test** | **Sensitivity** | | | | | |
| --- | --- | --- | --- | --- | --- | --- | --- | --- |
|  |  |  | **Test applied** | **80%** | **95%** | **Test for group effect** | **80%** | **95%** |
| Hosseini et al. (2016) | 20 | 10 | one-sample t-test on regression slopes (1-sided) | d = 0.85 | d = 1.13 | mixed-ANOVA (2x4) | d = 1.53 | d = 1.92 |
| Kober et al. (2015) | 20 | 7 sessions | linear regression | d = 2.66 | d = 3.46 | mixed-ANOVA (2x7) | d = 1.70 | d = 2.10 |
| Kober et al. (2018) | 48 | 12 | one-sample t-test on regression slopes (1-sided) | d = 0.77 | d = 1.02 | independent t-test on regression slopes (1-sided) | d = 1.05 | d = 1.39 |
| Lapborisuth et al. (2017) | 22 | 22 | one-sample t-test (1-sided) | d = 0.55 | d = 0.73 | Independent t-test 1-sided | d = 1.10 | d = 1.45 |
| Lee et al. (2015) | 4 | 4 | one-sample t-test (1-sided) | d = 1.65 | d = 2.23 | independent t-test 1-sided | d = 3.98 | d = 5.52 |
| **Mean** | 22.8 | 11 |  | d = 1.3 | d = 1.71 |  | d = 1.87 | d = 2.48 |
| **Median** | 20 | 10 |  | d = 0.85 | d = 1.13 |  | d = 1.53 | d = 1.92 |

Comparison of sensitivity for tests reported by studies comparing the effect to baseline and sensitivity for hypothetical comparison with control group/condition. Note that in order to simplify the analysis, for some studies we performed the analysis for a different statistical test than the one originally reported, did not take into account correction for multiple comparisons, and assumed no sphericity violation and a high correlation among repeated measures for ANOVAs. Overall, these measures should have led to overestimation of statistical power/sensitivity of the studies.

# References

Gelman, A. (2018, March 15). You need 16 times the sample size to estimate an interaction than to estimate a main effect [Blog post]. Retrieved from <https://statmodeling.stat.columbia.edu/2018/03/15/need-16-times-sample-size-estimate-interaction-estimate-main-effect/#comment-685111/>

Hudak, J., Blume, F., Dresler, T., Haeussinger, F. B., Renner, T. J., Fallgatter, A. J., et al. (2017). Near-Infrared Spectroscopy-Based Frontal Lobe Neurofeedback Integrated in Virtual Reality Modulates Brain and Behavior in Highly Impulsive Adults. *Front. Hum. Neurosci.* 11, 1–13. doi:10.3389/fnhum.2017.00425.

Marx, A.-M. M., Ehlis, A.-C. C., Furdea, A., Holtmann, M., Banaschewski, T., Brandeis, D., et al. (2015). Near-infrared spectroscopy (NIRS) neurofeedback as a treatment for children with attention deficit hyperactivity disorder (ADHD)-a pilot study. *Front Hum Neurosci* 8, 1038. doi:10.3389/fnhum.2014.01038.

Ros, T., Enriquez-Geppert, S., Zotev, V., Young, K. D., Wood, G., Whitfield-Gabrieli, S., et al. (2020). Consensus on the reporting and experimental design of clinical and cognitive-behavioural neurofeedback studies (CRED-nf checklist). *Brain*. doi:10.1093/brain/awaa009.

Schönenberg, M., Wiedemann, E., Schneidt, A., Scheeff, J., Logemann, A., Keune, P. M., et al. (2017). Neurofeedback, sham neurofeedback, and cognitive-behavioural group therapy in adults with attention-deficit hyperactivity disorder: a triple-blind, randomised, controlled trial. *The lancet. Psychiatry* 4, 673–684. doi:10.1016/S2215-0366(17)30291-2.

Tufanaru, C., Munn, Z., Aromataris, E., Campbell, J., and Hopp, L. (2017). “Chapter 3: Systematic reviews of effectiveness.,” in *Joanna Briggs Institute Reviewer’s Manual.*, eds. E. Aromataris and Z. Munn (The Joanna Briggs Institute). Available at: https://reviewersmanual.joannabriggs.org/.

Wampold, B. E., Minami, T., Tierney, S. C., Baskin, T. W., and Bhati, K. S. (2005). The placebo is powerful: Estimating placebo effects in medicine and psychotherapy from randomized clinical trials. *J. Clin. Psychol.* 61, 835–854. doi:10.1002/jclp.20129.
